# CNT-integrated MoO_3_ nanoparticles for bifunctional electrocatalytic oxygen and hydrogen evolution reactions

**DOI:** 10.1039/d6ra06716f

**Published:** 2026-10-28

**Authors:** Abid Ali, Reemal Imtesal, Saneea Ata-ul-Haq, Muhammad Shakeel Alam, Wan Jefrey Basirun, Murat Kaleli, Salih Akyürekli, Amna Safdar, Munawar Iqbal, Kholoud S. Al-Mugren

**Affiliations:** a Department of Chemistry, Faculty of Science, Universiti Malaya Kuala Lumpur 50603 Malaysia; b Department of Chemistry, The University of Lahore 1-km Defence Road Lahore Pakistan shakeelpcc@gmail.com; c Department of Chemistry, Lahore College for Women University Lahore Pakistan; d Innovative Technologies Application and Research Center (YETEM), Süleyman Demirel University Isparta 32260 Turkey; e Department of Chemistry, University of Engineering and Technology Lahore Pakistan; f Department of Chemistry, Faculty of Science, University of Tabuk Tabuk 47913 Saudi Arabia; g Physics Department, College of Science, Princess Nourah bint Abdulrahman University Riyadh 11671 Saudi Arabia; h Zero Emission Technologies Innovation Center, University of Tabuk Tabuk 47913 Saudi Arabia

## Abstract

The escalating global demand for sustainable and clean energy urges the development of efficient electrocatalysts to overcome sluggish oxygen evolution reaction (OER) and hydrogen evolution reaction (HER) kinetics in overall water splitting. This study investigates the key role of interfacial engineering by integrating orthorhombic α-MoO_3_ nanoparticles with conductive carbon nanotubes (CNTs) to form a MoO_3_@CNTs nanocomposite and systematically compares it with pristine MoO_3_ to address conductivity-limited electrochemical performance. The underlying poorly conductive MoO_3_ is employed as a reference system to evaluate the impact of CNT incorporation on charge-transfer resistance, active-site accessibility, and overall bifunctional catalytic efficiency. The electrochemical evaluation trends in relation to overpotential, Tafel slopes, double-layer capacitance (*C*_dl_), electrochemically active surface area (ECSA), and charge-transfer resistance (*R*_ct_) are revealed with significantly improved outputs. Among the investigated materials, MoO_3_@CNTs performs remarkably better than pristine MoO_3_, achieving low overpotentials of 0.35 V for the OER (50 mA cm^−2^) and 0.33 V for the HER (−10 mA cm^−2^) in 1 M KOH, compared to 0.42 V and 0.40 V for MoO_3_, respectively. This study also reveals the enhanced electrocatalytic behavior of MoO_3_@CNTs, mainly due to improved electronic conductivity and increased active-site exposure induced by CNT integration, leading to reduced Tafel slopes (217 mV dec^−1^ for OER and 170 mV dec^−1^ for HER), lower *R*_ct_ (5.1 Ω *vs.* 10.3 Ω), and higher *C*_dl_ (26.07 mF cm^−2^*vs.* 19.85 mF cm^−2^) and ECSA (43.71 cm^2^*vs.* 30.43 cm^2^). These findings deepen the understanding of CNT-based interfacial engineering and its effect on MoO_3_ electrochemical activity and position MoO_3_@CNTs as a promising bifunctional catalyst for efficient and sustainable hydrogen production.

## Abstract 2

The escalating global demand for sustainable and clean energy urges the development of efficient electrocatalysts to overcome sluggish oxygen evolution reaction (OER) and hydrogen evolution reaction (HER) kinetics in overall water splitting.

## Graphical abstract

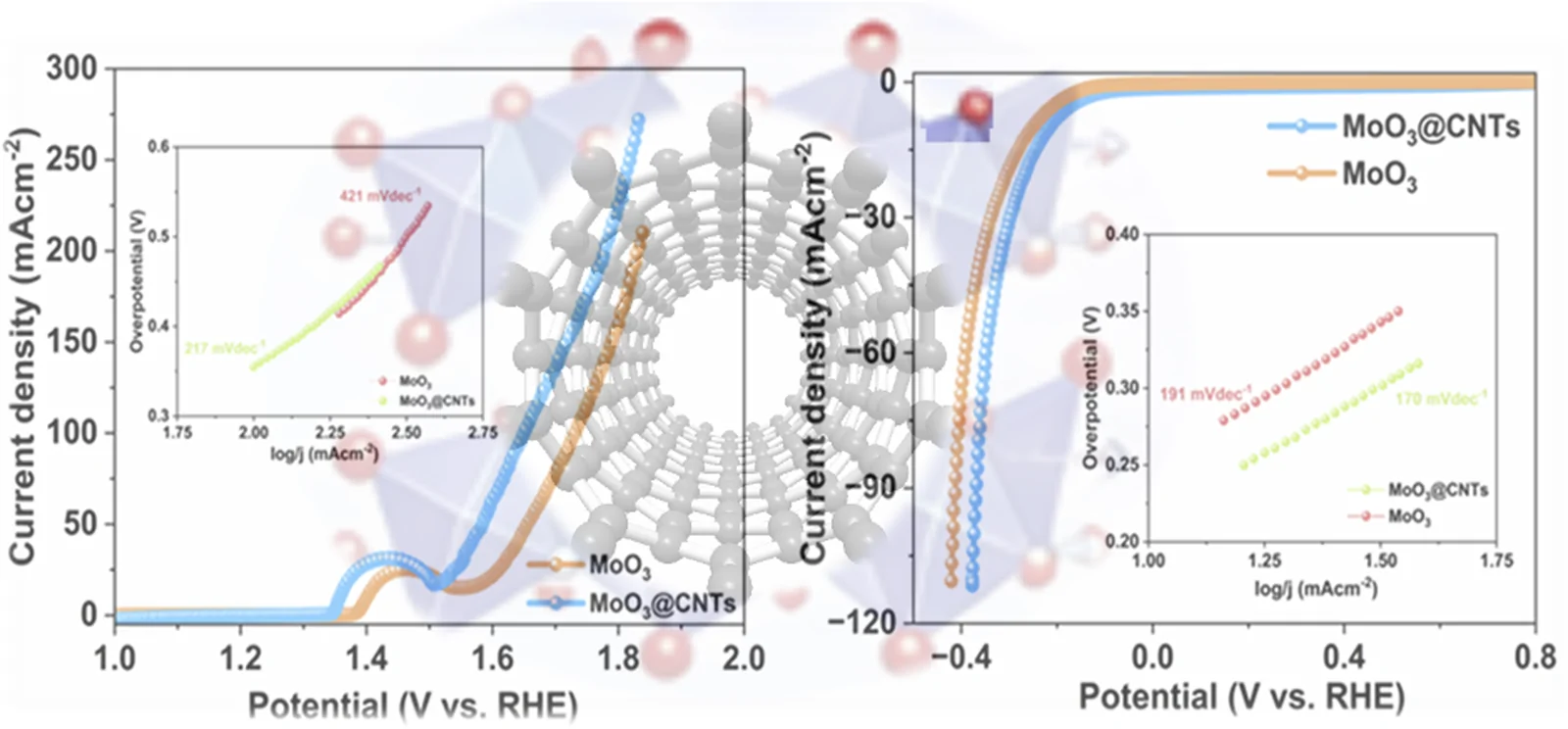

## 1. Introduction

The depletion of fossil fuel resources and the associated impact of greenhouse gases have intensified the demands of sustainable and carbon-neutral energy technologies.^1^ In this context, hydrogen (H_2_) has become one of the most interesting clean energy carriers due to its high gravimetric energy density, environmental compatibility and zero-emission combustion properties.^1–4^ Electrocatalytic water splitting using renewable electricity is one of the most promising hydrogen production technologies because of its sustainability, ease of operation, and scalability.^5–7^ Water electrolysis is a fundamental process that consists of two half-reactions: hydrogen evolution reaction (HER) on the cathode and oxygen evolution reaction (OER) on the anode.^8,9^ In the case of HER, the reaction occurs *via* relatively simple proton/electron transfer mechanisms, whereas the OER is a four-electron transfer process involving complicated oxygen-containing intermediates with slow reaction kinetics and is the major kinetic barrier for overall water splitting.^10–12^ Thus, the design of efficient bifunctional electrocatalysts that can promote both HER and OER is one of the key challenges for hydrogen production systems.

Currently, the most active and low-overpotential catalysts used are based on noble metals, namely, Pt for HER and RuO_2_/IrO_2_ for OER.^11–13^ Because of their high price, poor natural availability and poor long-term stability in practical operating conditions, however, they are severely limited in large-scale commercialization.^14–16^ Much research has thus been dedicated to investigating the development of earth-abundant and low-cost alternatives that are highly efficient and durable as catalysts. In particular, transition metal oxides (TMOs) have gained great interest over the past few years due to their versatile electronic structures, multiple oxidation states, structural stability, and extensive surface redox chemistry.^17–19^ Of particular interest are new electrocatalytic materials such as molybdenum trioxide (MoO_3_), which have a layered crystal structure, are non-toxic, possess a high theoretical capacitance and have favorable electrochemical properties.^20–22^ In terms of crystallography, MoO_3_ is known in α-, β-, and h-phases, of which the orthorhombic α-MoO_3_ is considered the most stable and electrochemically active phase, because of the structure of a layer of distorted MoO_6_ octahedra.^23–25^ This structure allows for easier intercalation of ions and encourages surface reactions with oxygen. However, MoO_3_ alone has intrinsic electronic conductivity, electrochemically active surface area and charge-transfer kinetics that are all intrinsically poor, and this comprehensive deficiency hinders the intrinsic electrocatalytic efficiency of MoO_3_.^22,24,26^

To overcome these limitations, coupling MoO_3_ with conductive carbon nanostructures has emerged as an effective strategy to simultaneously enhance charge transport and active-site utilization.^27–30^ Among them, carbon nanotubes (CNTs) are particularly advantageous due to their exceptional conductivity, high aspect ratio, structural robustness, and large accessible surface area.^31,32^ The interconnected CNT framework establishes continuous electron-percolation pathways, facilitates rapid ion diffusion, and provides abundant nucleation sites for uniform dispersion of MoO_3_ nanostructures, thereby mitigating agglomeration and maximizing active-site exposure.^33–37^ However, their tendency to aggregate due to strong van der Waals interactions necessitates controlled interfacial engineering to achieve homogeneous hybrid architectures.^38^ Reports of bifunctional overall water splitting of MoO_3_/CNT composites are limited, although MoO_3_-based composites with other conductive or heteroatom-modified supports (*e.g.*, NiCoP, Ni–NiO, activated carbon, Co_2_P_2_O_7_, Co_3_O_4_, MXene) have been reported for electrodes for electrochemical water splitting. In particular, the interfacial and kinetic behavior of MoO_3_, which does not include any secondary active phases or heteroatom doping, has not been systematically established upon incorporating CNTs as an active phase.

This gap is filled by the present study, which uniformly anchors MoO_3_ nanoparticles onto the CNT surface for the direct and controlled evaluation of the interfacial engineering effect on charge-transfer kinetics and active-site accessibility afforded by the CNT. It turns out that the resulting MoO_3_@CNTs electrode has significantly improved bifunctional electrocatalytic activity: 0.35 V (OER) at 50 mA cm^−2^ and 0.33 V (HER) at 10 mA cm^−2^, along with substantially reduced Tafel slopes (217 mV dec^−1^ for OER; 170 mV dec^−1^ for HER), and increased electrochemically active surface area (*C*_dl_ = 26.07 mF cm^−2^; ECSA = 43.71 cm^2^) and charge-transfer resistance (*R*_ct_ = 5.1 Ω). The results are complemented by an integrated study of the structural and electrochemical properties that shows that the synergy between MoO_3_ and CNTs overcomes the intrinsic conductivity and charge-transfer limitation of MoO_3_, providing a promising strategy for rational design of transition-metal-oxide-based bifunctional water-splitting catalysts in a single, consistently self-contained study.

## 2. Experimental section

### 2.1 Chemicals and materials

All chemicals and materials employed in this work were of analytical grade and used as received without further purification. Multiwalled carbon nanotubes (MWCNTs, >98% purity, O.D. × length = 6–13 nm × 2.5–20 µm, product no. 698849) were procured from Sigma-Aldrich, ammonium molybdate tetrahydrate, citric acid, ammonia solution, ethanol, methanol, nitric acid (HNO_3_), sulfuric acid (H_2_SO_4_), and hydrochloric acid (HCl) were procured from Sigma-Aldrich. Deionized (Milli-Q) water was used throughout all experimental procedures.

### 2.2 Preparation of functionalized CNTs

The functionalized carbon nanotubes (CNTs) were prepared *via* acid oxidation treatment as represented in step 1 of Scheme 1. Briefly, 0.2 g of CNTs were dispersed in a mixed acid solution of concentrated HNO_3_ and H_2_SO_4_ (1 : 3, v/v) and ultrasonicated for 1 h to improve the surface hydrophilicity and introduce oxygen-containing functional groups. The resulting suspension was stirred continuously at room temperature for 18 h and subsequently diluted with deionized water to a final volume of 100 cm^3^. The obtained functionalized CNTs were washed repeatedly with distilled water followed by ethanol washing until neutral pH was attained. Finally, the product was dried at 60 °C for 8 h in a hot-air oven.

**Scheme 1:**
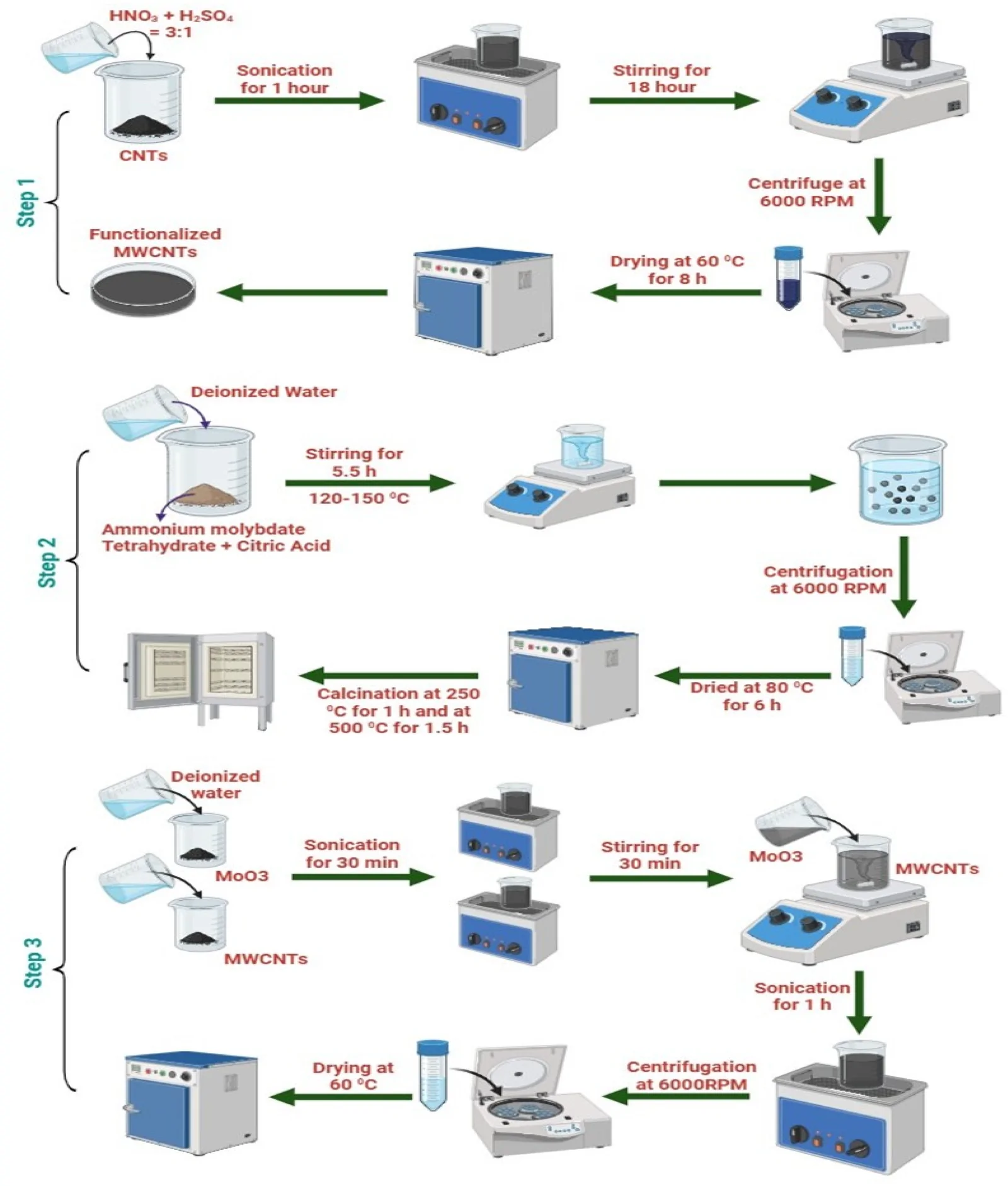
Schematic illustration of the (step 1) synthesis of functionalized MWCNTs, (step 2) synthesis of MoO_3_ by sol–gel method and (step 3) sono-chemical synthesis route for the fabrication of the MoO_3_@CNTs nanocomposite.

### 2.3 Synthesis of MoO_3_

A schematic diagram of the synthesis procedure for MoO_3_ nanoparticles is shown in step 2 of Scheme 1. Ammonium molybdate tetrahydrate was dissolved in DI water under continuous stirring at 120–150 °C. Citric acid was then introduced, followed by dropwise addition of NH_4_OH until neutral pH was obtained. The mixture was stirred for 5.5 h to form a homogeneous gel. The gel was centrifuged, washed, and dried at 80 °C for 6 h. The dried precursor was calcined at 250 °C for 1 h and subsequently at 500 °C for 90 min to obtain phase-pure orthorhombic α-MoO_3_ nanoparticles.

### 2.4 Synthesis of MoO_3_@CNTs composite

CNTs were functionalized with metal oxide nanoparticles using a sonochemical method as shown in step 3 of Scheme 1. To prepare the MoO_3_@CNTs composite, 0.15 g of functionalized MWCNTs was dispersed in 50 mL of distilled water and sonicated for 30 minutes to form a uniform suspension. Separately, 0.3 g of MoO_3_ nanoparticles synthesized *via* the citrate sol–gel method was dispersed in 25 mL of distilled water and sonicated for 30 minutes. The MoO_3_ solution was slowly added to the MWCNTs suspension under stirring, and the mixture was sonicated for 1 hour until a dark black colloidal paste formed. The precipitates were then centrifuged at 6000 rpm for 20 minutes, washed and then dried at 60 °C. The final dried composite weighed approximately 0.38 g. This composite material was then analyzed using linear sweep voltammetry (LSV) and cyclic voltammetry (CV) to investigate the oxidation process in the water-splitting reaction.

### 2.5 Material characterization

The prepared materials, MoO_3_, functionalized CNTs and MoO_3_@CNTs composite were subjected to X-ray diffraction (XRD) on a Rigaku Ultima IV diffractometer to analyze the crystalline phase and structural integrity. Fourier transform infrared spectroscopy (FTIR) was used to study the surface functional groups and chemical bonding properties. The optical absorption properties of the samples were studied by UV-visible spectroscopy with absorption transitions to assess the electronic transitions and optical properties. Scanning Electron Microscope (SEM) was used to examine the surface morphology and microstructural features. An energy-dispersive X-ray spectroscopy (EDX) system attached to the SEM system was also used to analyse the elemental composition and spatial distribution of the constituent elements within the sample.

### 2.6 Electrode fabrication and electrochemical measurements

The electrocatalytic water-splitting performance of MoO_3_ and MoO_3_@CNTs was evaluated using a Gamry Reference 3000 electrochemical workstation in a conventional three-electrode configuration. To investigate the OER and HER activities, the experiments were carried out in 1 M KOH and 0.5 M H_2_SO_4_ electrolytes, respectively. Initially, the working electrodes were prepared by dispersing each catalyst (MoO_3_ or MoO_3_@CNTs) in a water/Nafion mixture under ultrasonication to form a homogeneous ink. The resulting suspension was then drop-casted onto a freshly activated Ni-foam electrode (NF) and dried at 50 °C for 2 hours to obtain a uniform and stable catalytic layer. A platinum sheet and a double-junction Ag/AgCl electrode were used as the counter and reference electrodes, respectively. Cyclic voltammetry (CV) measurements were performed in the non-faradaic region at various scan rates for electrochemical surface area (ECSA) analysis. Linear sweep voltammetry (LSV) was recorded at a scan rate of 5 mV s^−1^ within the potential windows suitable for OER and HER evaluation, and all potentials were converted to the reversible hydrogen electrode (RHE) scale using the standard equation*E*_RHE_ = *E*_Ag/AgCl_ + 0.197 + 0.059pH

Electrochemical impedance spectroscopy (EIS) was used in the frequency range of 1 Hz to 10 kHz to examine the charge transfer resistance and interfacial electron transport properties of the catalyst. The Nyquist response showed that there was facilitated charge transfer at the electrode/electrolyte interface. Chronopotentiometry was performed at 10 mA cm^−2^ for 10 h to assess the electrochemical stability of the samples. The potential profile of the catalyst remained stable during the test with only slight variations, indicating stable charge transfer kinetics and electrochemical durability over extended operating lifetimes. The electrochemical measurements presented here were performed on well-prepared and characterized electrodes and were performed with standard electrochemical procedures; however, to minimize instrumentation time, it was not the goal of this study to statistically quantify the reproducibility of measurements across different sets of electrodes that were prepared independently. Triplicate measurements with error analysis will be used in the future to further enhance the statistical robustness of the reported electrocatalytic performance.

## 3. Results and discussions

### 3.1 Physical characterization

#### 3.1.1 Scanning electron microscopy (SEM)

SEM micrographs of the synthesized materials Fig. 1a–f provide detailed insight into the morphological characteristics and structural evolution of orthorhombic MoO_3_ across different magnification scales. At low magnification, densely packed MoO_3_ nanoparticles are observed forming irregular but interconnected agglomerated clusters, suggesting a nucleation-driven growth process accompanied by strong interparticle interactions. At higher magnification Fig. 1b–d, relatively uniform and well-dispersed nanoparticles with an average size of ∼100–150 nm are clearly observed. Such nanoscale features are highly beneficial for electrocatalytic applications, as they shorten ion diffusion pathways and increase the density of exposed active surface sites.^39^ The higher magnification images of the MoO_3_ particles show sharp-edged rod- and belt-like shapes with rough and defect-rich surfaces Fig. 1e and f, typical of orthorhombic α-MoO_3_.^40^ This anisotropic growth behavior is promoted by its layered crystal structure, which leads to a directionality of charge transport, and also enables access to many edge sites, which provide adsorption sites for important intermediates in both the HER and OER reactions in this structure (H*, OH*, OOH*) that promote improved catalytic reaction kinetics.^41,42^ The pristine α-MoO_3_ also presents plate-like nanoflower assemblies of well aligned microbelt, which also indicates high crystallinity and ordered self-assembly behavior.^43^

**Fig. 1:**
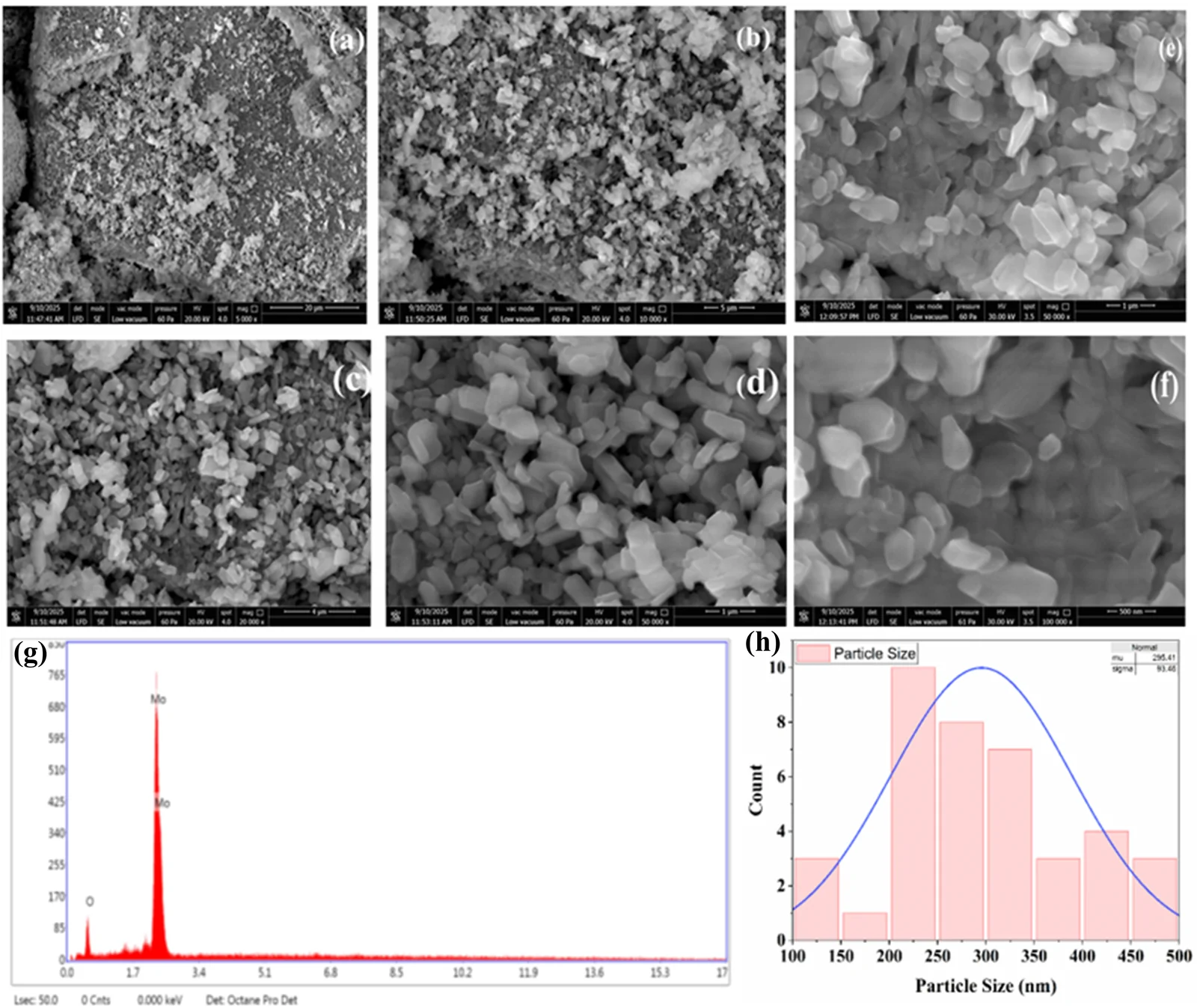
SEM micrographs of synthesized MoO_3_ nanoparticles at (a–c) lower, (d–f) higher magnifications, (g) EDX spectrum and (h) particle size histogram of MoO_3_ nanoparticles.

High phase purity as confirmed by EDS analysis in Fig. 1g confirms the absence of Mo impurities and the presence of O only. The elemental composition of the sample matches the literature-reported formula MoO_3_ with the slight deviations being due to oxygen deficiency and surface defect states corresponding to ∼28–35 at% Mo and ∼65–72 at% O. In the investigated sample, the Mo-rich signal was accompanied by oxygen distribution, thus indicating successful formation of MoO_3_ and minor variation in oxygen distribution indicating the presence of oxygen vacancies.^44,45^ These vacancies cause defect levels in the electronic structure, thereby increasing electrical conductivity and creating additional active sites that have great catalytic activity for electrocatalytic water splitting.^46^ Quantitative particle-size analysis, performed by measuring the diameters of 40 individual nanoparticles from the SEM micrographs Fig. 1f using ImageJ software, revealed a mean particle size of 262 ± 16 nm, consistent with the qualitative observation of the ∼500 nm size range noted above. The corresponding size-distribution histogram is presented in Fig. 1h.

The morphology of the MoO_3_@CNT composite in Fig. 2a–d shows that the intrinsic plate- and belt-like morphology of the MoO_3_ is preserved after its incorporation into CNTs, with significantly reduced agglomeration and improved dispersion. The CNT network not only provides continuous 3D conductive scaffolds around MoO_3_ nanospheres, but also facilitates good interfacial contact, avoids the restacking of particles, and supports the efficient transport of electrons, thereby significantly lowering the charge-transfer resistance and improving the electrochemical kinetics.^47,48^ Further homogeneous spatial distribution of both Mo and O was confirmed by EDS elemental mapping, which is visible as different colors in the results (Fig. 3a–e), where strong overlap of all elemental signals indicates successful integration of the CNTs, which did not phase-separate from the Mo–O matrix.^49^ Based on the quantitative elemental distribution, it is found that there are ∼69% Mo, ∼13% O and ∼18% C, with carbon acting as the conductive backbone and MoO_3_ as the redox-active catalytic centers.^16^ This uniform elemental dispersion allows for efficient electron percolation, low resistive losses and a high electrochemically active surface area.^50^ Importantly, this hierarchical MoO_3_/CNT architecture significantly improves the electrocatalytic water splitting performance by synergistically combining high surface area, oxygen vacancy-induced active sites, and excellent conductivity, which results in faster charge transfer kinetics, better electrolyte diffusion, lower polarization resistance and good stability.^51^

**Fig. 2:**
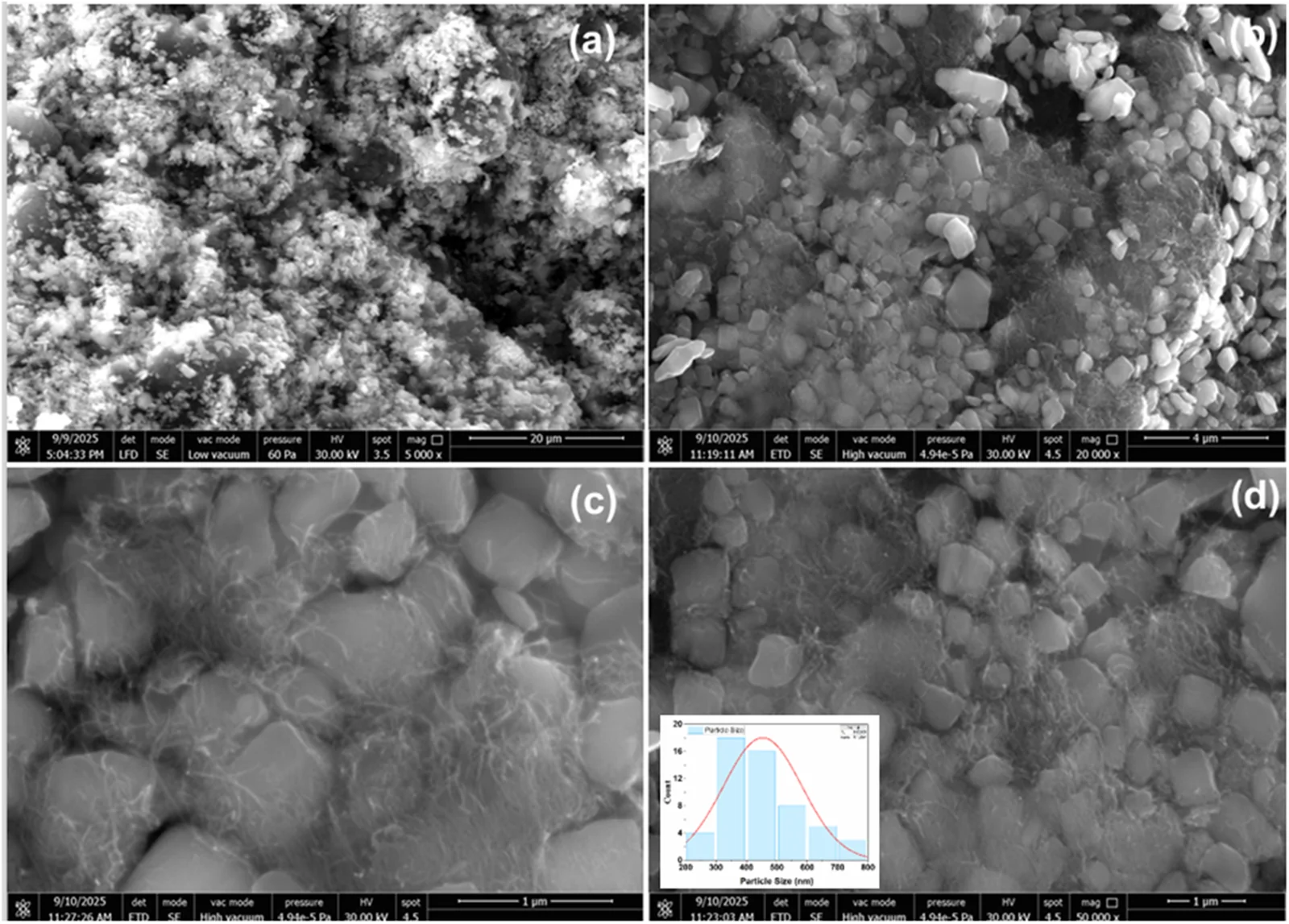
SEM micrographs of synthesized MoO_3_@CNTs composite at (a and b ) lower and (c and d) higher magnifications.

**Fig. 3:**
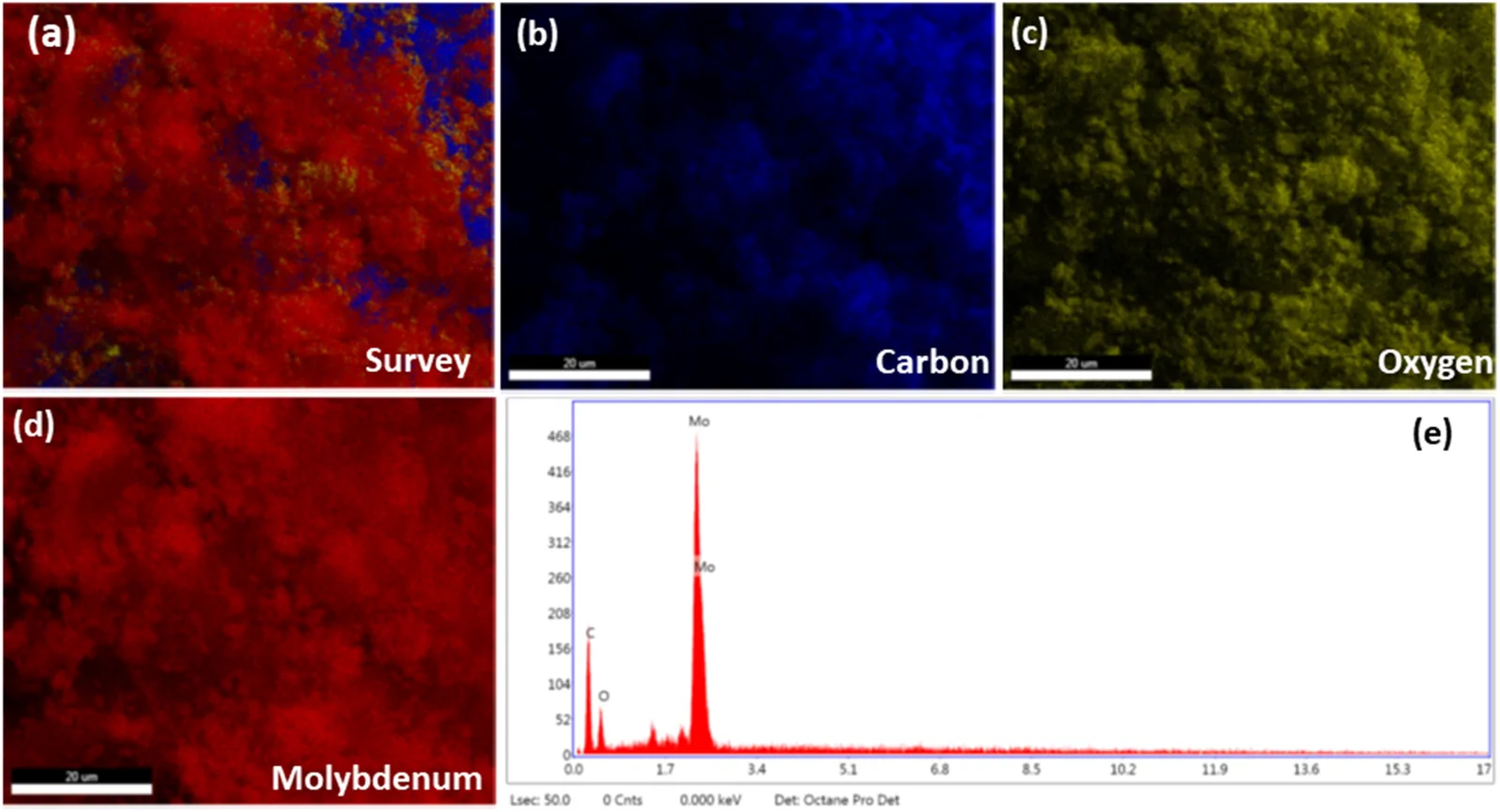
(a) Elemental mapping of the MoO_3_@CNTs nanocomposite revealing elemental composition with (b) carbon, (c) oxygen and (d) molybdenum and (e) EDS spectrum of MoO_3_@CNTs .

#### 3.1.2 Transmission electron microscopy

The TEM micrograph of the MoO_3_@CNTs composite (Fig. 4) reveals discrete, electron-dense nanoparticles, attributed to MoO_3_, distributed along the CNT sidewalls and inter-tube junctions, rather than forming large, phase-segregated aggregates. While this morphological observation is consistent with improved MoO_3_ dispersion on the CNT scaffold, as corroborated by the SEM and EDS analyses, it does not by itself resolve atomic-scale lattice continuity at the MoO_3_–CNT interface. Independent and convergent evidence for genuine structural and electronic integration between the two phases is instead provided by (i) the reduction in MoO_3_ crystallite size from ∼45 to ∼32 nm upon CNT incorporation (Scherrer analysis, Section 3.1.3), indicating that CNTs constrain MoO_3_ crystal growth; (ii) the systematic lattice parameter/unit-cell volume expansion (∼1.24%) observed by XRD (Section 3.1.3); (iii) the emergence of a Mo^4+^ component in the XPS Mo 3d spectrum, indicative of interfacial charge transfer (Section 3.1.6); and (iv) the shift in Raman D/G band positions relative to pristine MWCNT literature values (Section 3.1.4). Taken together, these complementary observations support the formation of a structurally and electronically coupled MoO_3_–CNT heterostructure. We note that direct atomic-resolution confirmation *via* HRTEM lattice-fringe imaging was not accessible within the scope of the present study and is identified as a valuable direction for future, more detailed structural investigation of this system.

**Fig. 4:**
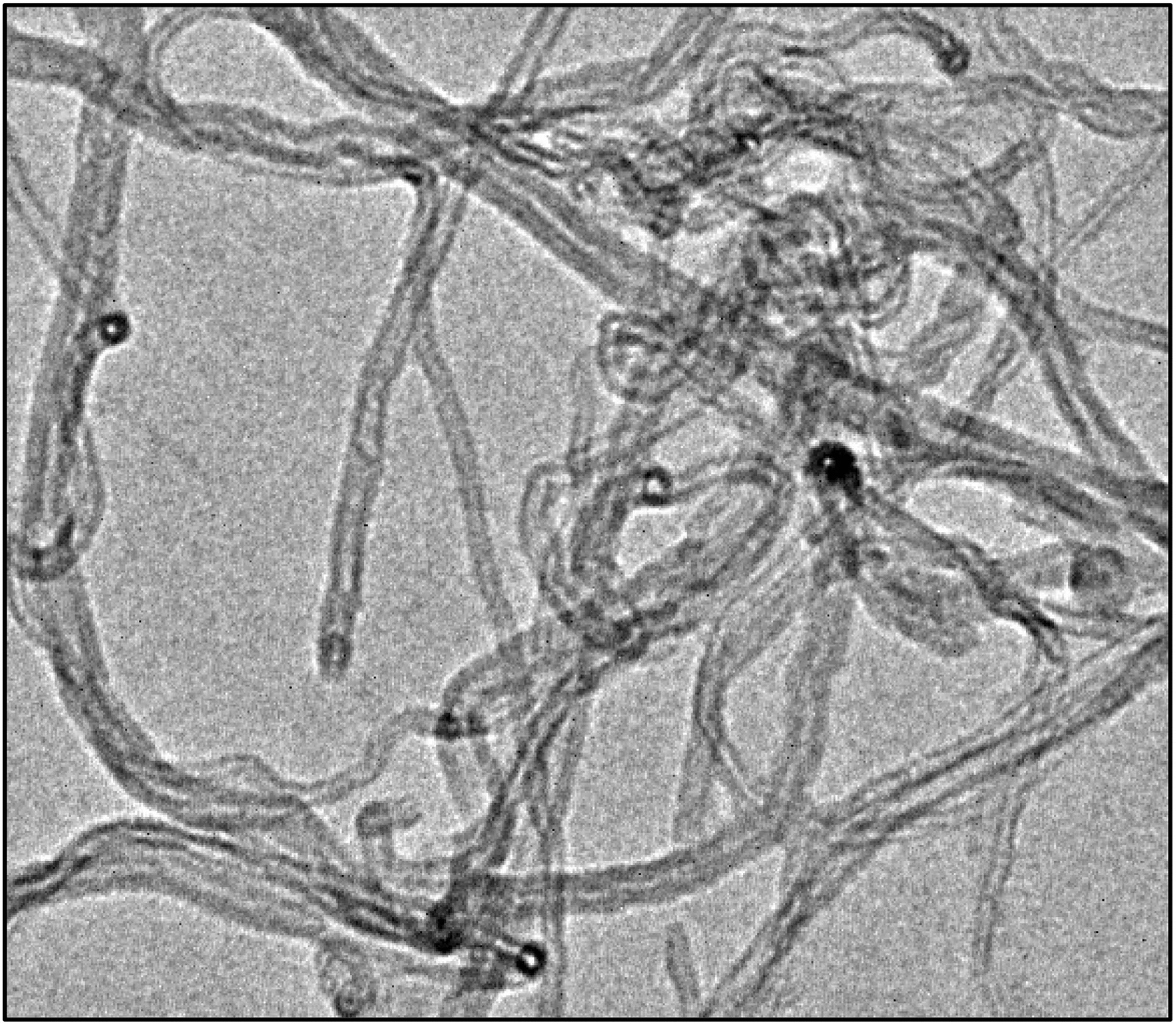
TEM micrograph of the MoO_3_@CNTs nanocomposite.

#### 3.1.3 X-ray diffraction (XRD)

Fig. 5 presents the XRD patterns of pristine MoO_3_ and the MoO_3_@CNT nanocomposite, respectively. The diffraction peaks located at 2*θ* values of ∼12.78°, 23.33°, 25.67°, 27.31°, 29.05°, 33.70°, 35.48°, 39.01°, 45.80°, 48.62°, 50.15°, and 58.70° are indexed to the (020), (110), (040), (021), (130), (111), (041), (060), (230), (140), (040), and (060) planes of orthorhombic α-MoO_3_ (ref. 52 and 53) (JCPDS no. 05-0508), confirming the high crystallinity and phase purity of the synthesized materials.^54–56^ The intense reflections (020), (040), and (060) are characteristic of the anisotropic crystal with preferential growth along the *b*-axis of MoO_3_, as shown in Fig. 5. All the characteristic MoO_3_ reflections are preserved in the MoO_3_/CNT composite, while no phase change is observed. Furthermore, the slight peak broadening and reduced diffraction intensity of the composite indicate a decrease in crystallite size, lattice distortion and strong interfacial interaction between the layers of MoO_3_ and CNTs. The mean crystallite sizes calculated from the Scherrer equation were around 45 and 32 nm for the pure MoO_3_ and MoO_3_/CNT composite, respectively, indicating that the crystal growth was suppressed by the CNTs and resulted in the interconnected nanoscale structure that is beneficial for the enhanced electrochemical charge-transfer kinetics.^57^

**Fig. 5:**
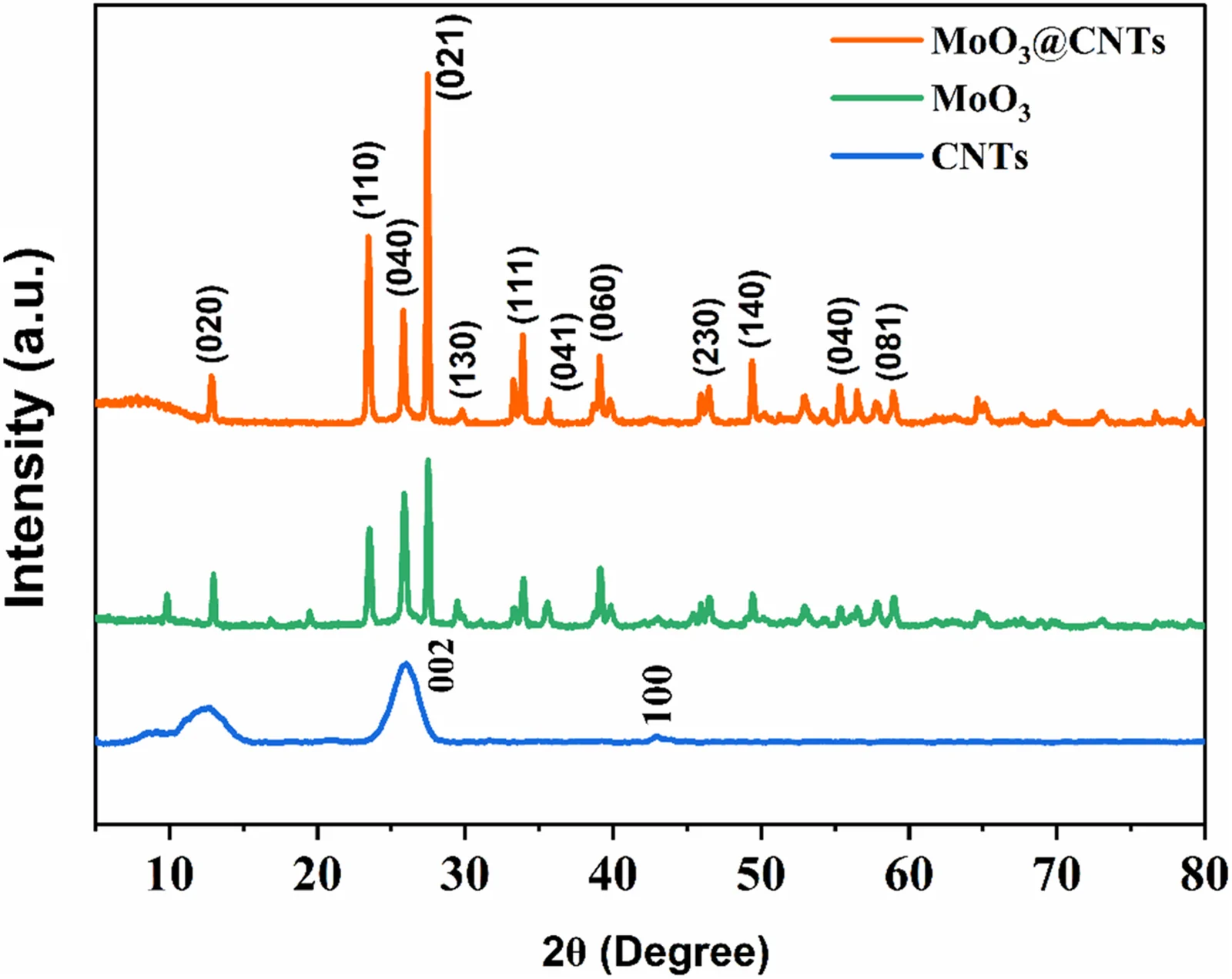
XRD patterns of pristine CNTs, pristine MoO_3_ and MoO_3_@CNTs composite.

The lattice parameters of pristine MoO_3_ and the MoO_3_@CNTs composite were calculated based on the indexed reflections in the XRD pattern, (020), (110), (040), (021), and (111) according to the orthorhombic unit-cell relation of MoO_3_, aiming to measure the quantitative structural changes after CNT incorporation. The calculated lattice parameters for pristine MoO_3_ (*a* = 3.933 Å, *b* = 13.672 Å, *c* = 3.681 Å) are in good agreement with the standard JCPDS card no. 05-0508 (*a* = 3.96 Å, *b* = 13.86 Å, *c* = 3.70 Å), confirming the phase purity of the synthesized α-MoO_3_. The characteristic MoO_3_ reflections were found to shift consistently along with other characteristic reflections downwards in 2*θ* upon incorporation of CNT, indicating an increase in the lattice parameter (*a* = 3.948 Å, *b* = 13.796 Å, *c* = 3.678 Å) and the overall increase in unit-cell volume of ∼1.24% (from 197.90 to 200.35 Å^3^). The expansion happens mainly along the *b*-axis (+0.90%) and the *a*-axis (+0.38%) sees a lesser increase with the *c*-axis having almost no change (−0.07%). This systematic lattice expansion is believed to be due to partial reduction of Mo^6+^ to Mo^4+^ at the interface of the MoO_3_ and CNT, as directly confirmed by XPS (Section 3.1.6); this phenomenon is believed to be caused by the larger ionic radius of Mo^4+^ compared to Mo^6+^ after incorporation. This observation is also in agreement with the variation of the oxygen distribution detected in EDX mapping (Section 3.1.1), and together represents multi-technique evidence for the formation of oxygen vacancies, and a meaningful structural/electronic coupling, between MoO_3_ and the CNT scaffold (Table 1).

**Table 1:** Calculated lattice parameters of MoO_3_ and MoO_3_@CNTs from indexed XRD reflections

Sample	*a* (Å)	*b* (Å)	*c* (Å)	Volume (Å^3^)
MoO_3_	3.933	13.672	3.681	197.90
MoO_3_@CNTs	3.948	13.796	3.678	200.35

For the detailed comparative study of the structure, the XRD pattern of the pristine CNTs was obtained, as well as the XRD pattern of the MoO_3_@CNTs composite and the pure MoO_3_ powder, as shown in Fig. 5. The characteristic graphitic interlayer stacking plane reflection (002) is seen as a sharp, intense reflection at 2*θ* ≈ 26°, whereas a weaker reflection is observed at 2*θ* ≈ 43° and assigned to the (100) in-plane graphitic lattice reflection. An extra broad hump at 2*θ* ∼ 12°–14° suggests a small contribution of randomly orientated/turbostratic carbon, which is a common feature of MWCNTs. No new crystalline phase and no impurity formation as a result of composite synthesis was observed as all the reflections observed in the composite pattern are consistent with JCPDS card no. 05-0508 for the orthorhombic α-MoO_3_ phase. In particular, one can see that there are two areas of maximal overlap between the pristine CNT pattern and the composite pattern: (i) the broad low-angle CNT carbon feature (marked ∼12°–14°) and the MoO_3_(020) reflection; (ii) the sharp and intense CNT(002) reflection (marked ∼26°) and the MoO_3_(040)/(021) reflections in the composite. The overlap causes the pattern of MoO_3_@CNTs to show a significantly increased relative intensity of the (021) reflection, which represents indirect diffraction-based proof of the intimate structural integration of the crystallites of MoO_3_ and the CNT scaffold. This finding corroborates with the SEM/EDX elemental mapping (Section 3.1.1), the D/G band analysis from Raman spectroscopy (Section 3.1.4), and the C 1s XPS spectrum (Section 3.1.5), which all suggest that the CNT network exists in the composite, with good interfacial coupling.

#### 3.1.4 Fourier transform infrared (FTIR)

Fourier-transform infrared (FTIR) spectra of f-MWCNTs, MoO_3_, and the MoO_3_/f-MWCNT composite (Fig. 6). Exhibit distinct and well-resolved vibrational features. The f-MWCNTs show a band at 1470 cm^−1^ assigned to graphitic C=C (sp^2^) stretching, with weak bands near 2020 and 2170 cm^−1^ attributed to defect-related sp-hybridized carbon species (C≡C/C≡N) introduced during functionalization.^58,59^ MoO_3_ displays a strong terminal Mo=O stretching mode at ∼1010 cm^−1^, along with bands at ∼827 cm^−1^ corresponding to asymmetric/symmetric stretching vibrations of the Mo–O–Mo lattice, confirming the orthorhombic phase. Broad absorptions at ∼3430 and ∼1630 cm^−1^ arise from O–H stretching and H–O–H bending of surface-adsorbed water.^60,61^ In the composite, the emergence and slight shift of the ∼824 cm^−1^ band indicate modified Mo–O coordination environments induced by strong interfacial interaction with f-MWCNTs, confirming successful formation of the hybrid MoO_3_/CNT architecture.^62^ This shift confirms strong electronic coupling at the heterointerface, consistent with improved charge-transfer behavior observed electrochemically. Consequently, the optimized interfacial electronic structure facilitates more efficient adsorption/activation of key intermediates (*H, *OH, *O), thereby accelerating proton–electron transfer kinetics and enhancing overall HER and OER performance.

**Fig. 6:**
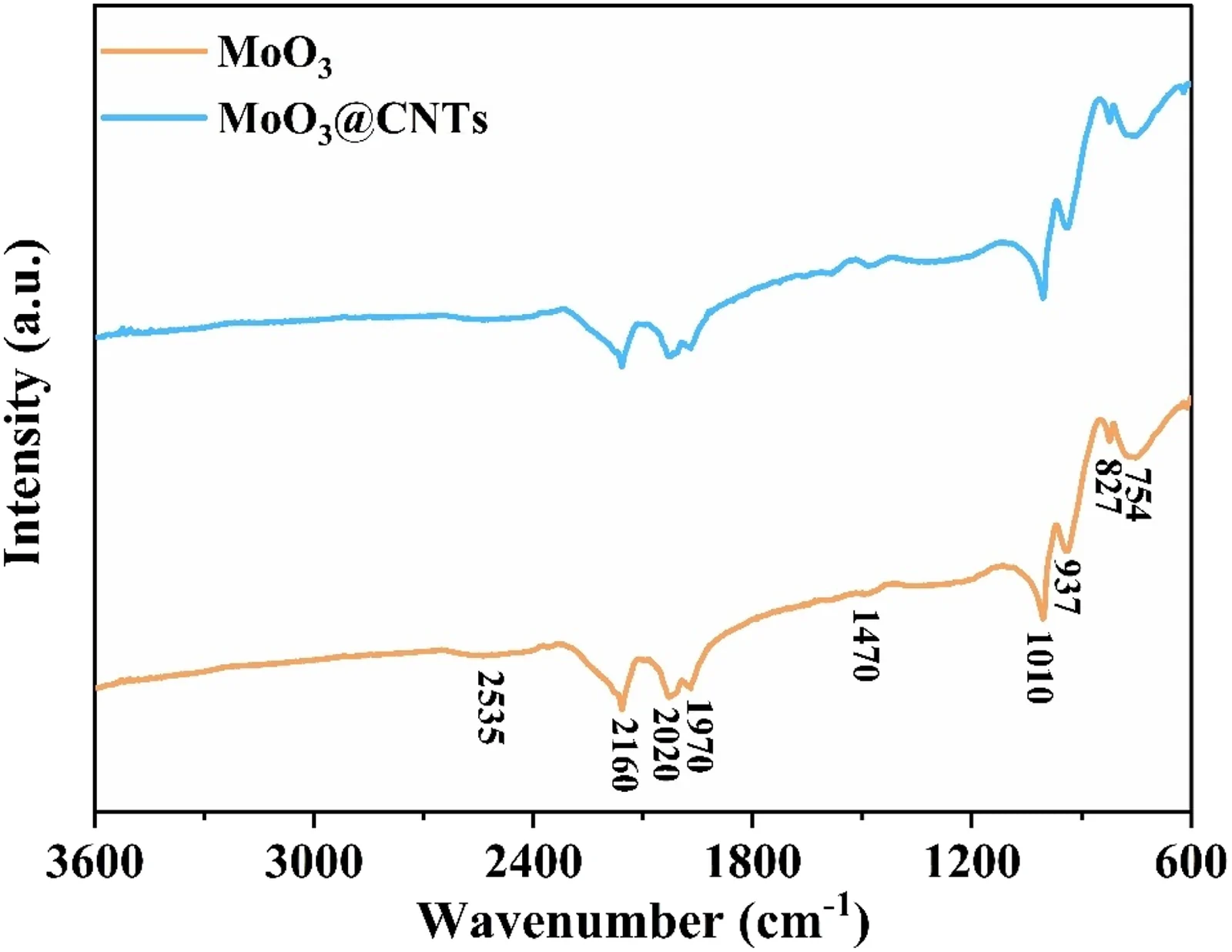
FT-IR analysis of MoO_3_ and MoO_3_@CNTs showing characteristic peaks in specific regions.

#### 3.1.5 Raman spectroscopy

The Raman spectrum of the MoO_3_@CNTs composite (Fig. 7) exhibits characteristic α-MoO_3_ lattice modes at 150, 280, 338, 658, 812, and 990 cm^−1^, corresponding to Mo–O–Mo bending, Mo=O stretching, and related lattice vibrations. In addition, two well-resolved and clearly separated bands are observed at 1334 and 1531 cm^−1^, corresponding to the D band (disorder-induced mode) and G band (E_2g_ graphitic mode) of sp^2^-hybridized carbon, respectively, confirming the presence of the CNT framework within the composite.^63–65^ A further second-order overtone (2D band) at 2674 cm^−1^ and a D + G combination mode at 2911 cm^−1^ are also identified, both characteristic of graphitic carbon materials and consistent with the D/G band assignment. The positions of the D and G bands show a modest downshift relative to typical literature values for pristine MWCNTs (∼1350 and ∼1580 cm^−1^)^66,67^ which is attributed to interfacial strain and charge-transfer interactions between the CNTs and the MoO_3_ lattice, consistent with the structural and electronic coupling evidenced by XRD and XPS analyses (Sections 3.1.2 and 3.1.5).

**Fig. 7:**
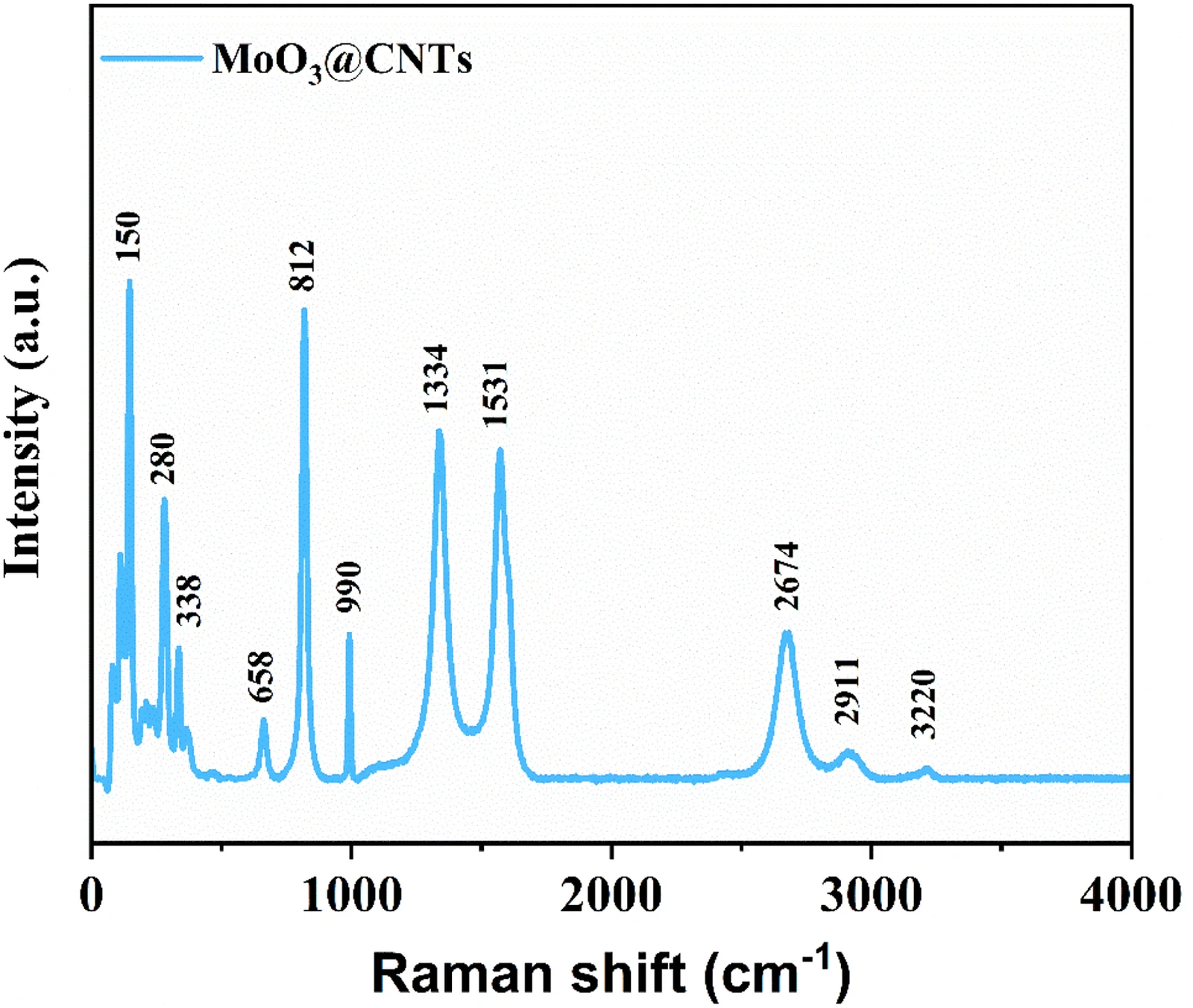
Raman spectrum of the MoO_3_@CNTs composite, showing characteristic α-MoO_3_ lattice modes (150–990 cm^−1^) and the resolved D (1334 cm^−1^), G (1531 cm^−1^), 2D (2674 cm^−1^), and D + G (2911 cm^−1^) carbon bands.

These observations are confirmed by a good degree of agreement with the FTIR analysis. The FTIR spectra show the same Mo–O and Mo–O–Mo vibrations at ∼986–1006 cm^−1^ and ∼812–853 cm^−1^, respectively, as well as a distinct graphitic C=C stretching mode at ∼1586 cm^−1^. The strong similarity between the two spectra substantiates that Raman and FTIR probe the same bonding environment, thus confirming the existence of the intact α-MoO_3_ lattice along with a well-integrated CNT network. The observed minor differences between the two techniques are attributed to electronic redistribution and strong coupling at the MoO_3_–CNT heterointerface rather than structural distortion.^37^ Importantly, this interfacial vibrational coupling, directly influences the electrocatalytic properties of the composite. Structures with the preserved Mo–O framework possess intrinsic redox-active sites necessary for oxygen evolution; meanwhile, the CNT network provides efficient electron transport pathways to facilitate charge transfer within the Mo–O surface. The synergistic structural-electronic integration has a positive effect on reducing kinetic barriers and therefore improving the HER and OER activity of the MoO_3_/CNT electrocatalyst.^44^

#### 3.1.6 X-ray photoelectron spectroscopy (XPS)

The surface elemental composition, presence of oxygen vacancies, and the electronic interaction between MoO_3_ and CNTs were directly confirmed by X-ray photoelectron spectroscopy (XPS) analysis of the MoO_3_@CNTs composite as shown in Fig. 8. C 1s spectrum (Fig. 8a): the high-resolution C 1s spectrum was deconvoluted into four components. The main peak at ∼284.6 eV is attributed to the sp^2^ hybridized C=C bonding of the graphitic CNT framework and the shoulder peak at ∼285.6 eV is attributed to the sp^3^ hybridized (defect/disordered) carbon. The minor component (represented by the peak at ∼286.8 eV) is assigned to oxygen-functionalized defect sites (C–O/disorder-related carbon, “D-B”), and the weak satellite feature at ∼290.8 eV is indicative of the π–π* shake-up transition, a hallmark peak of the extended sp^2^ conjugated network and thus confirms the retention of the graphitic CNT structure within the composite.

**Fig. 8:**
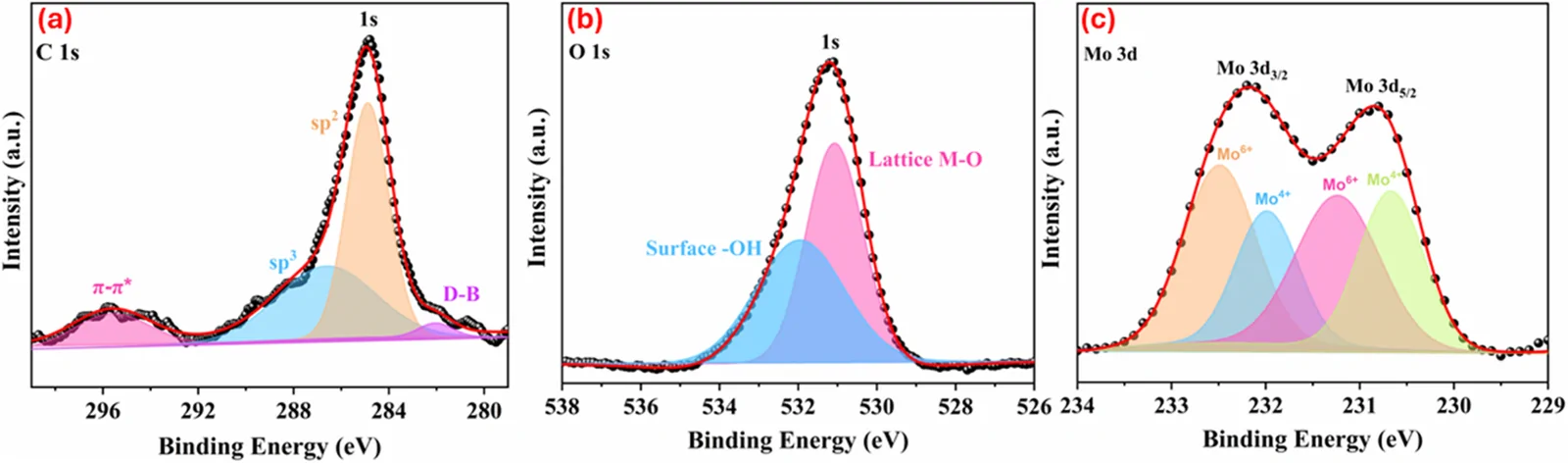
High-resolution XPS spectra of the MoO_3_@CNTs composite: (a) C 1s, (b) O 1s, and (c) Mo 3d.

O 1s spectrum (Fig. 8b), the O 1s spectrum has been divided into two parts. The first major peak at ∼530.9 eV is attributed to lattice bonding between metal and oxygen, thus indicating that the structure of α-MoO_3_ remains intact. The second peak, ∼532.1 eV, is associated with hydroxyl (–OH) groups and other surface-adsorbed oxygen species usually found on the surface of oxide nanostructures. Mo 3d spectrum (Fig. 8c): the Mo 3d spectrum shows the expected spin–orbit-split doublet (3d_5/2_ and 3d_3/2_) that was later deconvoluted into two different chemical states. The main doublet at ∼232.6 eV (Mo 3d_5/2_) and ∼235.7 eV (Mo 3d_3/2_) is due to the Mo^6+^ oxidation state of the stoichiometric MoO_3_. The doublet at ∼230.3 eV (Mo 3d_5/2_) and ∼233.4 eV (Mo 3d_3/2_) is considered the Mo^4+^ reduced oxidation state. The presence of the Mo^4+^, which coexisted with the dominant Mo^6+^ signal, directly provided spectroscopic evidence for the localized electron trapping at Mo centers, charge compensated by the loss of neighboring oxygen atoms, or the formation of oxygen vacancies. This is also supported by the O 1s spectrum, which has a surface-OH/defect-oxygen peak (∼532.1 eV), reflecting the oxygen coordination environment around this vacancy site.

The presence of oxygen vacancies in the MoO_3_@CNTs composite was further evidenced by the coexistence of both Mo 3d spectrum states, Mo^6+^ and Mo^4+^, and the presence of oxygen vacancies in the lattice-O 1s spectrum and the defect-O 1s spectrum, derived from the Mo 3d spectrum. In addition, the presence of the C 1s sp^2^ bonding energy level and the π–π* moiré satellite peak in the spectrum and the shifts in the Mo 3d bonding energies relative to bulk MoO_3_ (generally reported at 232.5/235.6 eV for Mo^6+^) supports the conclusion of strong interfacial electronic coupling between the MoO_3_ nanostructures and the CNT network, which is consistent with the vibrational shifts observed earlier in the Raman and FTIR spectra (Sections 3.1.3 and 3.1.4). This electronic redistribution at the MoO_3_–CNT heterointerface is anticipated to boost charge transfer kinetics and consequently the bifunctional MoO_3_–CNT composite will have superior HER/OER performance.

### 3.2 Electrochemical studies

#### 3.2.1 Linear sweep voltammetry (LSV)

Fig. 9a presents the *iR*-corrected linear sweep voltammetry (LSV) profiles of MoO_3_ and MoO_3_@CNTs recorded in 1 M KOH at a scan rate of 5 mV s^−1^. The measured potentials were referenced to the reversible hydrogen electrode (RHE) using*E*_RHE_ = *E*_SCE_ + 0.059 × pH + *E*_SCE0_and the oxygen evolution reaction (OER) overpotential was derived from the following equation.^68^*η* = *E*_RHE_ − 1.23 V

**Fig. 9:**
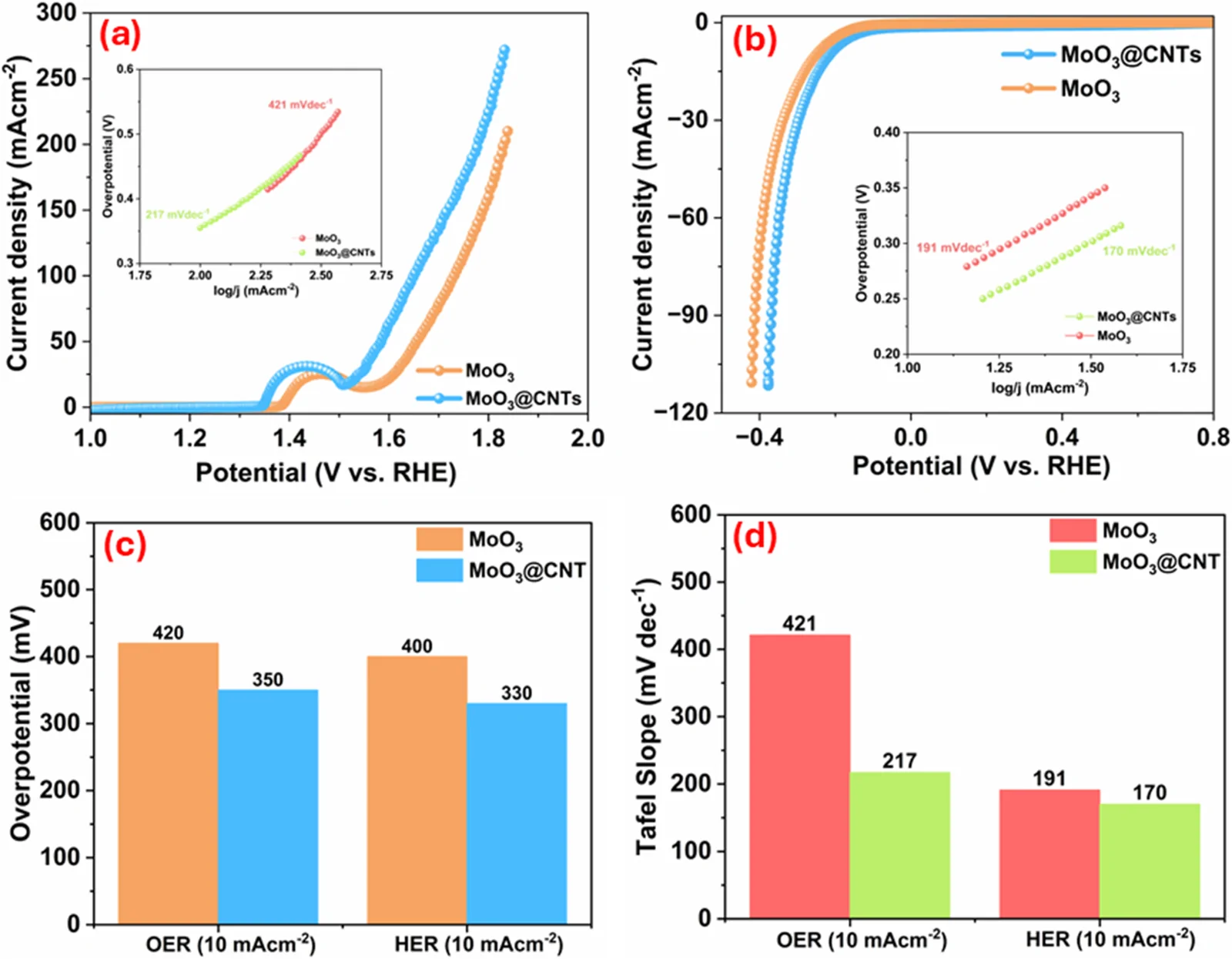
(a) LSV polarization curves and corresponding Tafel plots (inset) for OER, and (b) LSV polarization curves and corresponding Tafel plots (inset) for HER, comparing pristine MoO_3_ and MoO_3_@CNTs, recorded on a Ni-foam electrode in 1 M KOH electrolyte at a scan rate of 5 mV s^−1^ against Ag/AgCl reference. Bar graphs of (c) overpotentials at 50 mA cm^−2^ for OER and at 10 mA cm^−2^ for HER, and (d) Tafel slopes for OER and HER, for pristine MoO_3_ and MoO_3_@CNTs.

The overpotential for water oxidation of MoO_3_@CNTs is 350 mV at 50 mA cm^−2^ compared with 420 mV for pristine MoO_3_, indicating that a lower energetic cost is needed for water oxidation. The composite also shows a cathodically shifted onset potential, which means that the onset of the OER process happens at a more negative potential and the barrier for the thermodynamic formation of oxygen intermediates is reduced. The reaction kinetics were quantified by Tafel analysis in the form of *η* = *a* + *b* log *j*, where the Tafel slope ‘*b*’ is the potential increment needed to increase the current density by a factor of 10.^69^ Linear fitting resulted in Tafel slopes of 421 mV dec^−1^ for MoO_3_ and 217 mV dec^−1^ for MoO_3_@CNTs, indicating a significant improvement in charge-transfer kinetics when the CNTs were integrated. The lesser slope directly reflects the enhanced interfacial electron transfer and better reaction kinetics.

The synergistic improvement in electrocatalysis is due to both MoO_3_ and the conductive CNT network, which together provide improved electronic conductivity, increased electrochemically accessible active sites and accelerated transport of OH^−^ and oxygenated intermediates (OH*, O*, OOH*). All effects taken together have made MoO_3_@CNTs a much more effective OER electrocatalyst than pristine MoO_3_.^70^ The electrocatalytic activity of pure MoO_3_ and the MoO_3_/MWCNTs composite for the oxygen evolution reaction (OER) was systematically investigated by linear sweep voltammetry (LSV) in 1 M KOH alkaline electrolyte with a scan rate of 5 mV s^−1^. The polarization curve of MoO_3_/MWCNTs composite is shown with a high OER activity than MoO_3_ as seen in the polarization curve (Fig. 9a), where the anodic current density increases rapidly. In order to achieve the benchmark value of geometric current density of 50 mA cm^−2^ (Fig. 9c), the MoO_3_/MWCNTs composite needs only a potential of 350 mV, whereas the pure MoO_3_ electrocatalyst requires 420 mV. To further understand the OER reaction kinetics, Tafel plots were generated from the corresponding LSV (Fig. 9a (inset) and 9d). The Tafel slope of the MoO_3_/MWCNTs composite is significantly lower at 217 mV dec^−1^, in contrast to 421 mV dec^−1^ for bare MoO_3_. This large reduction suggests that the high conductivity of the MWCNTs has a dramatic impact on the electron transfer kinetics at the interface, and on the energetics of the catalytic pathway for water oxidation.

The bifunctional property of the synthesized materials was then evaluated, through the study of the hydrogen evolution reaction (HER) in a similar experimental setup. The cathodic LSV polarization curves (Fig. 9b) indicate that the MoO_3_/MWCNTs composite shows better performance than the pristine MoO_3_ in terms of current densities at lower applied cathodic potentials. In particular, Fig. 9c shows that the composite needs an overpotential of 330 mV for the current density of 10 mA cm^−2^, which is a significant 70 mV lower than MoO_3_ (400 mV). Moreover, the HER kinetics were analyzed using extracted Tafel slopes (Fig. 9b (inset) and 9d). Compared with pristine MoO_3_, whose Tafel slope is 191 mV dec^−1^, the MoO_3_/MWCNTs electrode provides an enhanced Tafel slope of 170 mV dec^−1^. Overall, the comparative electrochemical metrics confirm that integrating MoO_3_ with the MWCNT network fundamentally resolves the inherent poor electrical conductivity of pristine metal oxides. The 1D carbon matrix acts as a highly efficient electron transport highway, prevents the agglomeration of active materials, and maximizes the exposure of electrochemically active sites, culminating in superior bifunctional electrocatalytic activity for both OER and HER.

The MoO_3_@CNTs HER and OER overpotentials were then compared to literature-reported values of the standard commercial catalysts, *i.e.*, 20% Pt/C (HER) and RuO_2_ and IrO_2_ (OER). The HER overpotential (*η*_10_ = 40 mV, Tafel slope = 35.14 mV dec^−1^) of the commercial Pt/C is significantly lower compared to the MoO_3_@CNTs (*η*_10_ = 330 mV). It is noteworthy, however, that the MoO_3_@CNTs overpotential (*η*_10_ = 350 mV) is very comparable to that of the commercial benchmarks RuO_2_ (*η*_10_ = 327 ± 10 mV, Tafel slope = 67 mV dec^−1^) and IrO_2_ (*η*_10_ = 335 mV, Tafel slope = 97 mV dec^−1^) differing by only 15–23 mV. This indicates that MoO_3_@CNTs is a promising non-precious-metal catalyst for the OER half-reaction, with OER activity that is comparable to state-of-the-art noble-metal oxide catalysts, and with potential advantages of low cost, good earth abundance, and good scalability, as a candidate for practical alkaline water electrolyzers. It is recognized that direct experimental comparison with the present work, under identical experimental conditions, between the nature of the Pt/C and RuO_2_/IrO_2_ would yield a more stringent comparison and would be useful for the future (Table 2).

**Table 2:** Comparison of HER and OER overpotentials (*η*_10_) and Tafel slopes of the present MoO_3_@CNTs catalyst with commercial noble-metal-based benchmark electrocatalysts (Pt/C, RuO_2_, and IrO_2_)

Catalyst	HER, *η*_10_ (mV)	OER, *η*_10_ (mV)	Tafel slope (mV dec^−1^)	Ref.
Commercial 20% Pt/C	40	—	35.14	71
Commercial RuO_2_	—	327 ± 10	67	72
Commercial IrO_2_	—	335	97	73

Bare Ni-foam electrodes are well known to exhibit negligible intrinsic catalytic activity for HER and OER, functioning mainly as an inert current-collecting substrate. Pristine, unmodified CNTs likewise show only weak intrinsic electrocatalytic activity, typically requiring much higher overpotentials and larger Tafel slopes than transition-metal-oxide catalysts due to the lack of well-defined redox-active sites. Although direct control measurements under our specific experimental conditions were not feasible within the revision timeframe, the markedly lower overpotentials and Tafel slopes obtained for MoO_3_@CNTs relative to reported values for bare carbon substrates support the conclusion that the observed activity arises predominantly from the MoO_3_–CNT heterostructure. We acknowledge this as a limitation and plan to include direct control measurements in future work.

#### 3.2.2 Cyclic voltammetry

The Fig. 10a and b present the cyclic voltammetry (CV) curves of MoO_3_ and MoO_3_@CNTs, respectively, recorded in 1 M KOH electrolyte within the non-faradaic potential region of 0–0.20 V *versus* Ag/AgCl at different scan rates. Both electrodes exhibited nearly symmetric capacitive responses with quasi-rectangular features, indicating dominant electric double-layer capacitive behavior. Such electrochemical characteristics strongly influence the HER and OER catalytic activities by governing the accessibility of electrochemically active sites and the interfacial charge-transfer process. The scan rate progressively increased and the current density also increased in both electrodes, which indicated a fast ion diffusion and low diffusion resistance at the electrode/electrolyte interface. The MoO_3_@CNTs composite showed a significantly increased CV area and current density compared with pristine MoO_3_. The current density on the pristine MoO_3_ was increased from about −0.3/0.2 mA cm^−2^ at 5 mV s^−1^ to almost −1.5/+1.5 mA cm^−2^ at 70 mV s^−1^, while the MoO_3_@CNTs electrode showed remarkably high current densities reaching about −3.2/+3.5 mA cm^−2^ at 80 mV s^−1^. This finding shows that the MoO_3_@CNT composite has a higher electrochemically active site density than MoO_3_. Such enhanced electrochemical behavior can be attributed to two major factors: (i) improved electrical conductivity arising from the interconnected CNT conductive network and (ii) increased electrochemically active surface area resulting from improved dispersion of MoO_3_ active domains and suppression of particle agglomeration. The former will be further discussed in the EIS analysis, while the latter was verified through ECSA calculations.^74^ To further clarify the enhanced electrocatalytic performance of the MoO_3_@CNTs composite, the electrochemically active surface area (ECSA) was estimated using the following relations:
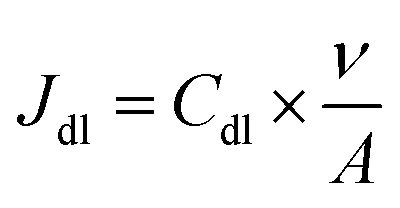

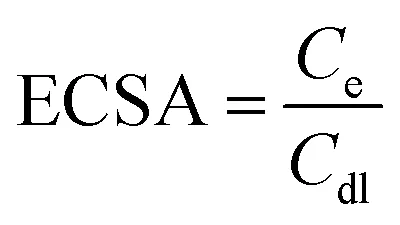
where *J*_dl_ represents the double-layer charging current density, *C*_dl_ denotes the non-faradaic double-layer capacitance, *ν* is the scan rate, *A* corresponds to the geometrical area of the working electrode, and *C*_e_ represents the specific capacitance of the electrolyte, taken as 0.04 mF cm^−2^ for KOH electrolyte.^75^ From the linear fitting plots shown in Fig. 10c, the *C*_dl_ values were determined to be 19.85 and 26.07 mF cm^−2^ for MoO_3_ and MoO_3_@CNTs, respectively, corresponding to ECSA values of 30.43 and 43.71 cm^2^. Moreover, at a scan rate of 0.07 V s^−1^, the MoO_3_@CNTs electrode exhibited a capacitive current density of ∼2.8 mA cm^−2^, which is more than twice that of pristine MoO_3_ (∼1.3 mA cm^−2^). The results clearly show that the electrochemically active surface area of the MoO_3_@CNTs composite is much higher and that the number of active sites that are uncovered is higher. This enhancement can be explained by the uniform distribution of MoO_3_ on the conductive CNT framework, which results in avoiding aggregation of active particles and facilitates electrolyte penetration to the electrode surface. This hierarchical composite architecture therefore offers better surface-to-volume ratios and better accessibility of the interfaces, which results in efficient electrocatalytic reactions.

**Fig. 10:**
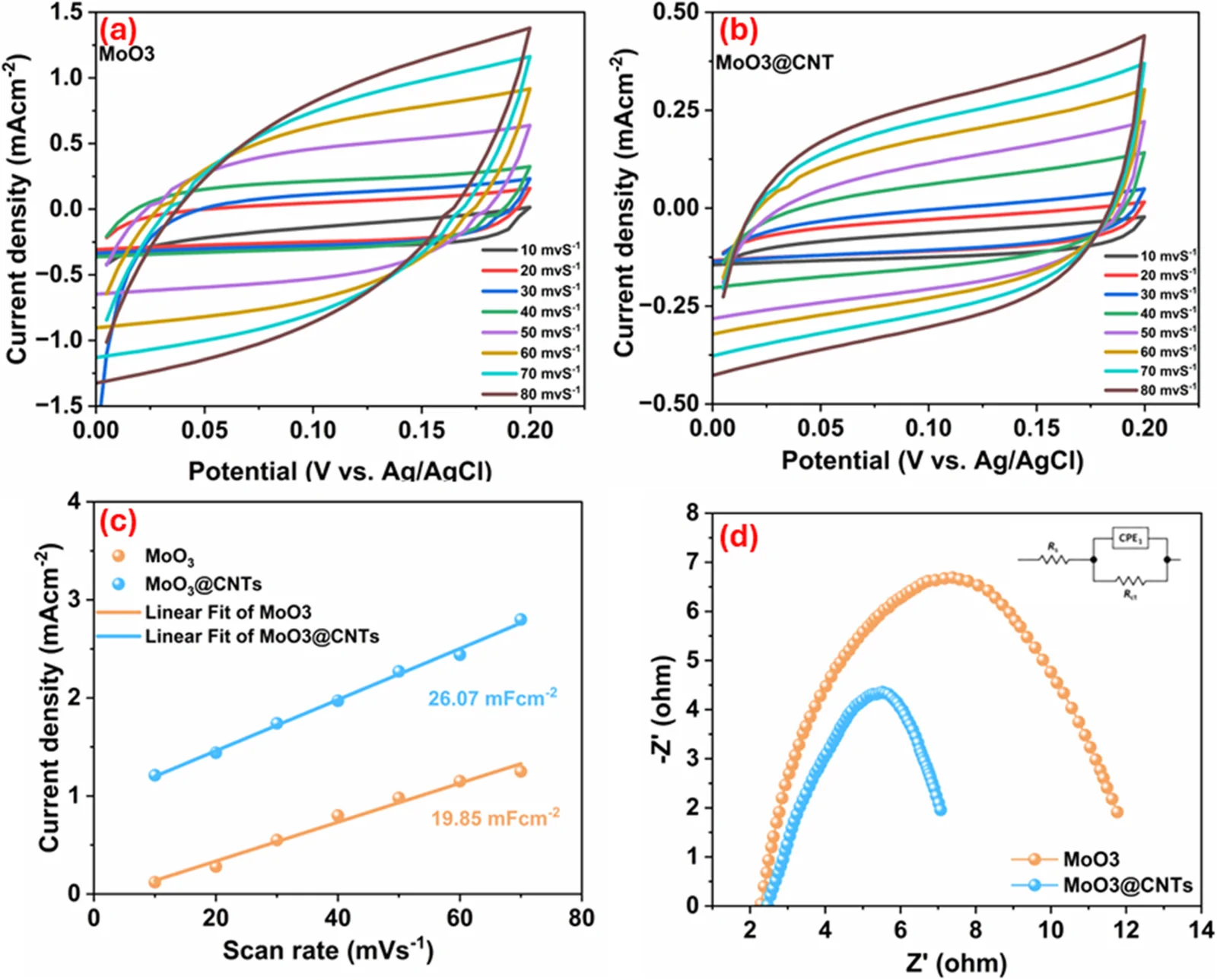
Cyclic voltammograms of (a) MoO_3_ and (b) MoO_3_@CNTs at different scan rates in 1 M KOH; (c) current density *vs.* scan rate for *C*_dl_ estimation; and (d) Nyquist plots (EIS) with fitted equivalent circuits, showing reduced charge-transfer resistance for MoO_3_@CNTs.

#### 3.2.3 Electrochemical impedance spectroscopy (EIS)

The electrochemical resistive characteristics of the electrodes were further verified by electrochemical impedance spectroscopy (EIS). The Nyquist plots in Fig. 10d indicated both electrodes had a semi-circle shape in the high-frequency region with a linear slope in the low-frequency region. The high-frequency semicircle is attributed to the interfacial charge-transfer resistance (*R*_ct_), while the linear low-frequency portion is related to the ion diffusion properties in the electrolyte/electrode interface. The equivalent circuit model was used to calculate the charge-transfer resistance values, which were found to be ∼10.3 Ω and ∼5.1 Ω for pristine MoO_3_ and the MoO_3_@CNTs composite, respectively. The solution resistance (*R*_s_) values were almost constant (∼2.3–2.5 Ω) for both electrodes, however. The significantly decreased semicircle diameter and the decreased *R*_ct_ of MoO_3_@CNTs electrode clearly indicate improved interfacial electron-transfer kinetics and reduced charge-transport resistance. This optimized conductivity is attributed to the interconnected CNT network, which creates efficient electron-conduction pathways and promotes the migration of charges across the multi-layered electrode architecture.^74^

In summary, it can be observed that the results from the three analyses, namely CV, ECSA, and EIS, are very clear to show that the incorporation of CNTs has an effective in enhancing the electrochemically active surface area and the charge-transfer characteristic of the MoO_3_ electrode. Synergistic interaction between conductive CNT networks and catalytically active MoO_3_ domains increases the accessibility of the electrolyte, the electron-transfer kinetics and the number of electrochemically accessible active sites.^76^ This results in an electrochemical performance of the MoO_3_@CNTs composite superior to that of pristine MoO_3_ and an HER/OER electrocatalytic performance that is better than both pristine MoO_3_ and CNTs (Table 3).

**Table 3:** Electrochemical parameters of overall water splitting for MoO_3_ and MoO_3_@CNTs

Electrode	Overpotential (*η*) (mV)		Tafel slope (mV dec^−1^)		*C*_dl_ (mF cm^−2^)	ECSA (cm^2^)	*R*_s_ (Ω)	*R*_ct_ (Ω)
	OER	HER	OER	HER				
MoO_3_	420	400	421	191	19.85	30.43	2.3	10.3
MoO_3_@CNTs	350	330	217	170	26.07	43.71	2.5	5.1

#### 3.2.4 Stability of electrocatalyst

From all the above results (Fig. 11), MoO_3_@CNTs exhibits superior stability during continuous chronoamperometric operation in 1 M KOH at a constant current density of 10 mA cm^−2^ for 10 h, showing only a slight variation in current response. The composite electrode retains ∼84% of its initial activity after prolonged electrolysis, confirming excellent operational durability and structural robustness. This enhanced stability is attributed to the strong interfacial coupling between MoO_3_ and the conductive CNT network, which stabilizes active sites, suppresses degradation, and maintains efficient charge transport during long-term operation.

**Fig. 11:**
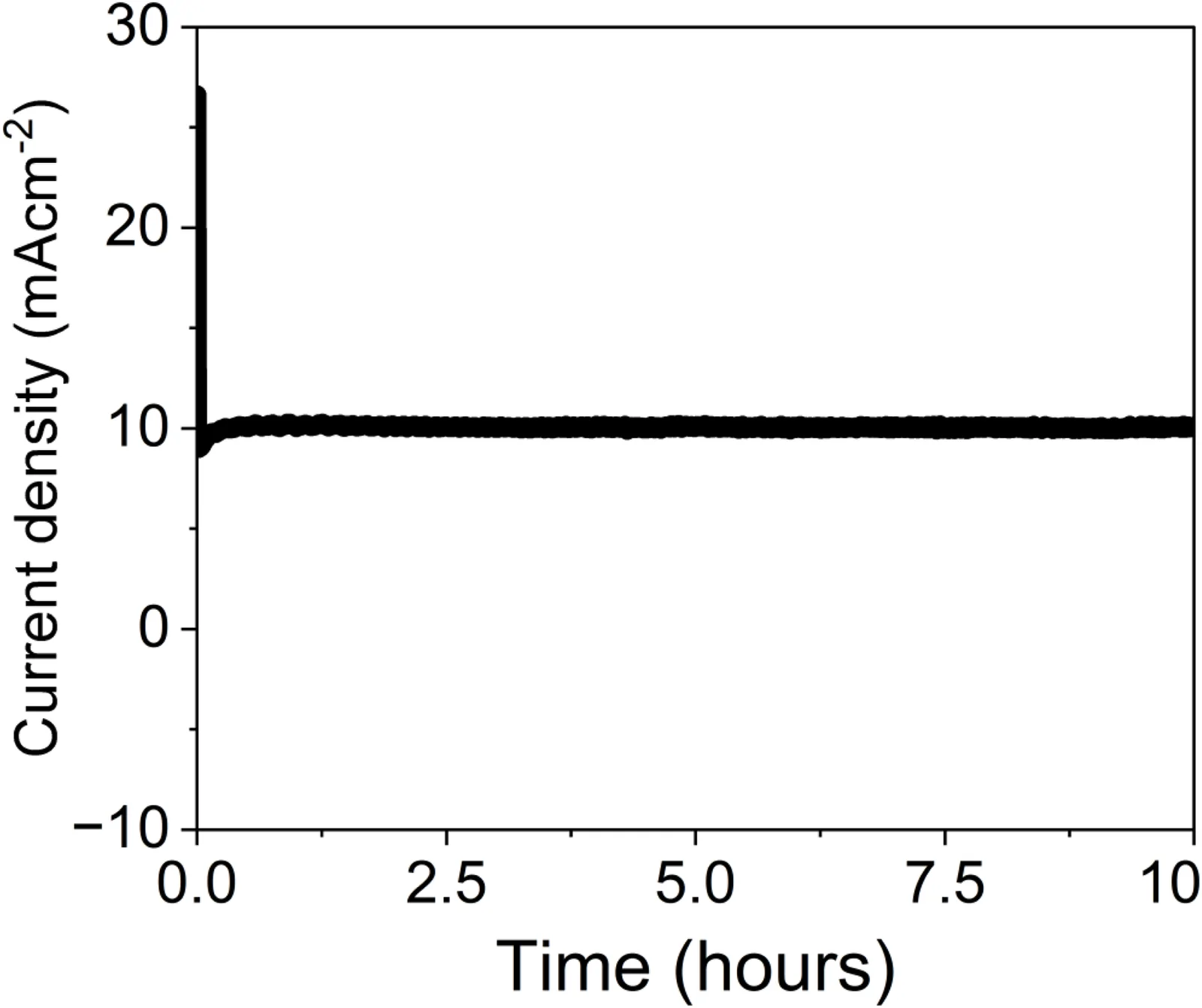
Chronoamperometric stability curve of the MoO_3_@CNTs electrode at a constant current density of 10 mA cm^−2^ for 10 h in 1 M KOH electrolyte.

Therefore, it can be concluded that CNT incorporation effectively improves both the electrocatalytic performance and stability of MoO_3_ toward alkaline water-splitting applications. For further enhancement of long-term durability and practical applicability, integration with highly conductive and porous substrates (*e.g.*, Ni foam, carbon cloth, or carbon paper) may provide a promising direction for optimizing the MoO_3_@CNTs system.

The overpotentials and Tafel slopes of the MoO_3_@CNTs are comparatively higher than those of the recently reported multi-component bifunctional electrocatalysts, such as Ni_3_S_2_@MoO_3_@Co_3_O_4_, MoO_3_–NiO@graphitic nitride, or Fe–NiCoP–MoO_3_, as shown in Table 4. It should be noted, however, that the most successful systems listed above are almost exclusively ternary or quaternary, hetero-structured systems, which generally involve multi-step doping, co-precipitation or heteroatom incorporation methods that increase the synthetic complexity and expense. The present MoO_3_@CNTs system, however, is a relatively straightforward two-component composite system that is prepared using a single-step, cost-effective, and scalable synthesis process, employing earth-abundant, non-precious metal precursors.

**Table 4:** Comparative electrochemical performance of MoO_3_@CNTs based nanocomposite electrodes for OER and HER studies

Electrode material	Overpotential (*η*) (mV)		Tafel slope (mV dec^−1^)		Current density (mA cm^−2^)		*C*_dl_ (mF cm^−2^)	Ref.
	OER	HER	OER	HER	OER	HER		
Ni_3_S_2_@MoO_3_@Co_3_O_4_	248	185	36.3	99.6	10	10	—	77
MoO_3_–NiO@graphitic nitride	198	92	96	94	10	10	—	78
MoO_3_/g-C_3_N_4_	187.6	139.3	126.2	120.2	10	10	30.5	79
CrNiCoO_4_/MoO_3_	279	260	45.31	—	100	50	24.18	80
Fe–NiCoP–MoO_3_	253	65	58.1	64	50	10	21.15	81
Co_2_P_2_O_7_@MoO_3_	227	58.7	70.9	59.1	50	10	19.5	82
MoO_3_/Ni–NiO	347	62	60	59	100	10	68.96	83
MoO_3_	350	387	47	146	10	10	4.96	84
Co_3_O_4_/MoO_3_/g-C_3_N_4_	206	125	60	94	10	10	1.57	85
Ti_3_C_2_@MoO_3_	190	91	59	34	10	10	—	86
α-MoO_3_/g-C_3_N_4_	188	139	120	126	10	10	112.5	87
MoO_3_	420	400	421	191	10	10	19.85	This work
MoO_3_@CNTs	350	330	217	170	10	10	26.07	This work

More impressively, the overpotential is seen to reduce from 420 mV (OER) and 400 mV (HER) to 350 mV (OER) and 330 mV (HER), respectively, after CNT integration, and the Tafel slope is seen to drop significantly from 421 to 217 mV dec^−1^ (OER), and from 191 to 170 mV dec^−1^ (HER), respectively, after CNT integration. The near two-fold increase in Tafel slope is an indication of significant improvement in reaction kinetics as confirmed by the XPS (Section 3.1.5) and Raman/FTIR (Sections 3.1.3 and 3.1.4) analyses, which suggests that the conductive CNT network provides enhanced charge-transfer pathways. Also, the comparison of the electrochemically active surface area (electrochemical and double-layer) of the composite and MoO_3_ alone revealed a substantially higher electrochemically active surface area (∼31%) of the composite, thus providing direct evidence for the synergistic interaction between MoO_3_ and CNT. The absolute performance in the present work is not yet comparable to the most optimized multi-metal heterostructures from 2024 to 2026; however, the internal evidence clearly indicates that CNTs have led to an enhancement of the reaction kinetics, the mechanism of which is well supported and the costs of which are minimal, thus creating a platform for bifunctional catalysis that is simple and structurally straightforward. The performance difference from more complex systems is indicative of a clear and promising path toward improvements in the future, such as heteroatom doping, defect engineering, or additional hybridization with additional transition metals in the secondary component, rather than a limitation of the underlying MoO_3_–CNT design concept.

## 4. Conclusion

Although the synthesis of the MoO_3_/CNTs synergistically combined electrocatalysts has resulted in great improvements in bifunctional electrocatalytic activity, some shortcomings must be pointed out. Long-term stability of the MoO_3_@CNTs composite in extreme alkaline conditions and at high current densities needs to be investigated further to ensure that the composite is suitable for practical use, as the carbon matrix may degrade over time or the MoO_3_ may dissolve under certain conditions. Moreover, their overpotentials for OER (0.35 V) and HER (0.33 V) are still significantly higher than those of state-of-the-art benchmark catalysts based on noble metals. The optimal design of interface engineering is still in an early stage and can be further optimized by introducing transition metal dopants (*e.g.*, Ni, Co, Fe) into the MoO_3_ lattice to further tune the electronic structure, and by exploring the 3D porous carbon architecture to improve mass transport and structural robustness. Moreover, *in situ*/*operando* characterization methods could be used to better understand the dynamics of the active sites during the electrocatalytic process for designing even more efficient and durable non-noble metal electrocatalysts.

## Conflicts of interest

The authors declare no conflict of interests.

## Supplementary Material

RA-016-D6RA06716F-s001

## Data Availability

The raw and processed data required to reproduce these findings, including structural characterizations and electrochemical measurements, are available from the corresponding author upon reasonable request. Supplementary information (SI): CV curves and LSV polarization curves of bare NF and CNTs@NF for OER in 1 M KOH. See DOI: https://doi.org/10.1039/d6ra06716f.
