# Supplementary material for: CNT-integrated MoO3 nanoparticles for bifunctional electrocatalytic oxygen and hydrogen evolution reactions
Source: RSC Adv. 2026 Oct 28. Online ahead of print. doi: 10.1039/d6ra06716f (PMC13714923; doi:10.1039/d6ra06716f)
Supplement: Supplementary file 1 [file RA-OLF-D6RA06716F-s001.pdf]

Supplementary Information (SI) for RSC Advances.
This journal is © The Royal Society of Chemistry

CNT-Integrated MoO₃ Nanoparticles for Bifunctional Electrocatalytic Oxygen and Hydrogen Evolution Reactions

Abid Ali[1,2], Reemal Imtesal[3], Saneea Ata-ul-Haq[3], Muhammad Shakeel Alam[2], Wan Jefrey Basirun[1], Murat Kaleli[4], Salih Akyürekli[4], Amna Safdar[5], Munawar Iqbal[6], Kholoud S. Al-Mugren[7]

[1]Department of Chemistry, Faculty of Science, Universiti Malaya, Kuala Lumpur, 50603, Malaysia

[2]Department of Chemistry, The University of Lahore, 1-Km Defence Road, Lahore, Pakistan

[3]Department of Chemistry, Lahore College for Women University, Lahore

[4]Innovative Technologies Application and Research Center (YETEM), Süleyman Demirel University, Isparta 32260, Turkey

[5]Department of Chemistry, University of Engineering and Technology, Lahore, Pakistan

[6]Department of Chemistry, Faculty of Science, University of Tabuk, Tabuk, 47913, Saudi Arabia

[7]Physics Department, Science College, Princess Nourah bint Abdulrahman University, Riyadh, Saudi Arabia

---

Corresponding Author

***Muhammad Shakeel Alam**

Email: shakeelpcc@gmail.com

Department of Chemistry, The University of Lahore, 1-Km Defense Road, Lahore, Pakistan

1.1 Control Electrochemical Measurements (Bare NF and CNTs@NF)

To isolate and clarify the individual electrochemical contributions of the Ni foam (NF) substrate and the CNT scaffold, independent of the MoO₃ active phase, control LSV and CV measurements were performed on bare NF and CNTs@NF electrodes under identical conditions (1 M KOH, Ag/AgCl reference) to those used for MoO₃ and MoO₃@CNTs.

As shown in Fig. S1a, the bare NF electrode requires an overpotential of 480 mV to reach a current density of 50 mA cm⁻^{2}, while CNTs@NF reaches the same current density at 430 mV, reflecting the moderate conductivity enhancement provided by the CNT coating. Both control electrodes exhibit a small anodic hump at ~1.3 V vs. RHE, corresponding to the characteristic Ni(OH)₂ → NiOOH surface redox transition of Ni foam in alkaline electrolyte. Both control electrodes show markedly inferior activity relative to the MoO₃@CNTs composite (350 mV at 50 mA cm⁻^{2}; main text, Fig. 9).

CV measurements performed over a range of scan rates (10–70 mV s⁻^{1}; Fig. S1b, c) were used to extract the double-layer capacitance (C_{dl}) via the slope of the linear fit of current density vs. scan rate (Fig. S1d). The bare NF and CNTs@NF electrodes yield Cdl values of 0.666 and 2.267 mF cm⁻^{2}, respectively — substantially lower than that of the MoO₃@CNTs composite (26.07 mF cm⁻^{2}; main text, Section 3.2.2). These results confirm that the markedly larger electrochemically active surface area and the superior OER/HER performance of the composite arise predominantly from the incorporated MoO₃ active sites, synergistically supported by the conductive CNT network, rather than from the Ni foam substrate or CNT coating alone.

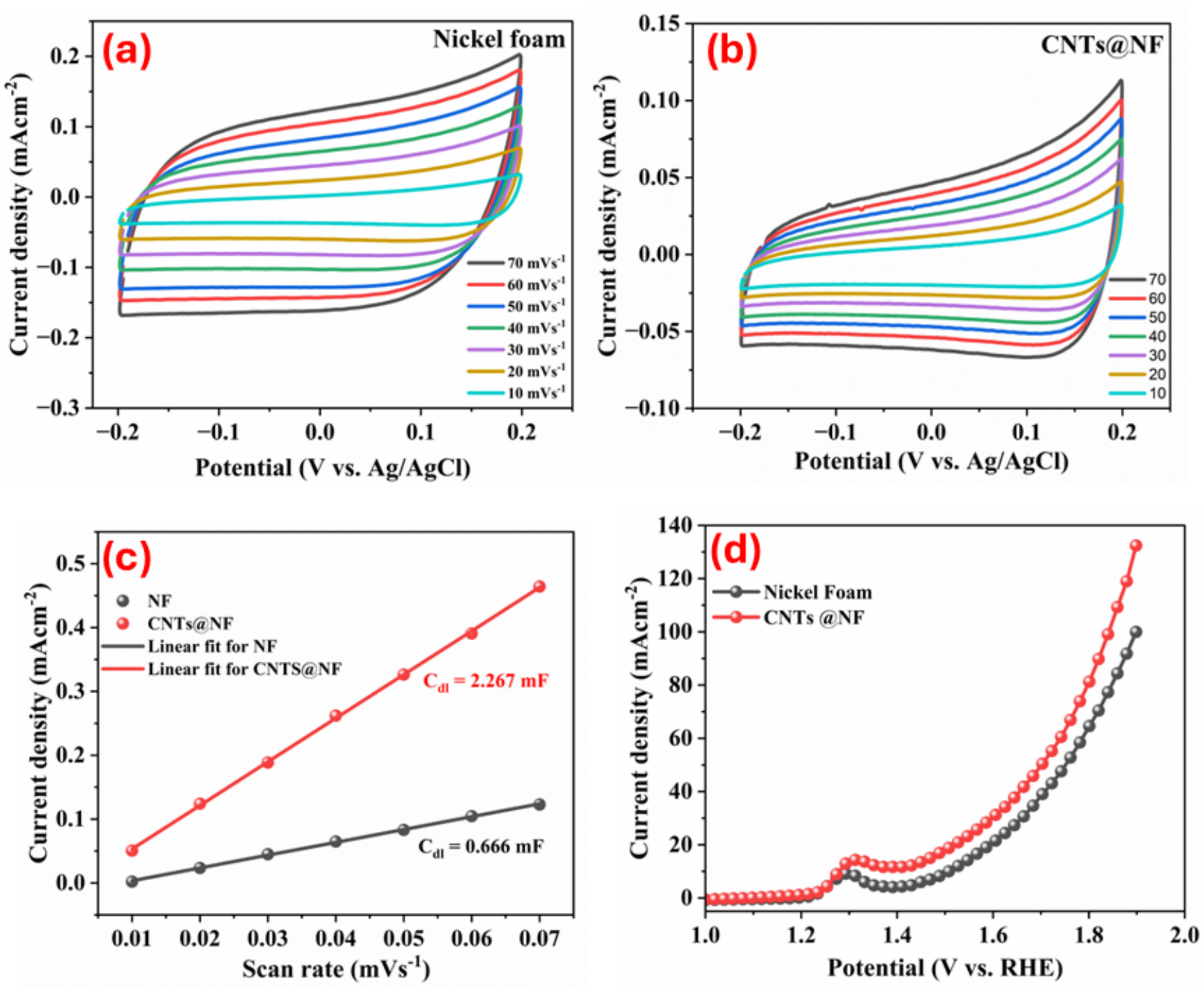

Fig. S1. **(a)** CV curves of bare Ni foam (NF) at scan rates of 10–70 mV s⁻^{1}. **(b)** CV curves of CNTs@NF at scan rates of 10–70 mV s⁻^{1}. **(c)** Linear fit of current density vs. scan rate for NF and CNTs@NF used to determine Cdl. **(d)** LSV polarization curves of bare NF and CNTs@NF for OER in 1 M KOH.
